# Breast Cancer Risk Assessment and Primary Prevention Advice in Primary Care: A Systematic Review of Provider Attitudes and Routine Behaviours

**DOI:** 10.3390/cancers13164150

**Published:** 2021-08-18

**Authors:** Sarah Bellhouse, Rhiannon E. Hawkes, Sacha J. Howell, Louise Gorman, David P. French

**Affiliations:** 1 Manchester Centre for Health Psychology, Division of Psychology and Mental Health, School of Health Sciences, Faculty of Biology, Medicine and Health, University of Manchester, Manchester M13 9PL, UK; rhiannon.hawkes@manchester.ac.uk (R.E.H.); david.french@manchester.ac.uk (D.P.F.); 2 Division of Cancer Sciences, Faculty of Biology, Medicine and Health, University of Manchester, Manchester Academic Health Science Centre, Manchester M13 9PL, UK; sacha.howell@nhs.net; 3 NIHR Greater Manchester Patient Safety Translational Research Centre, University of Manchester, Manchester Academic Health Science Centre, Manchester M13 9PL, UK; louise.gorman@manchester.ac.uk

**Keywords:** primary care, breast cancer, risk assessment, primary prevention, systematic review

## Abstract

**Simple Summary:**

There is growing international interest in adopting a risk-based approach to breast cancer screening, where an individual’s risk would inform screening practices. It has been suggested that primary care will contribute to the delivery of this service by conducting risk assessment and providing primary prevention advice. The aim of our review was to understand what primary care providers think and feel about performing these tasks by examining their attitudes and typical activity in clinical practice (routine behaviours). The results suggest that primary care providers mainly assess breast cancer risk by collecting family history information but feel less comfortable advising on risk-reducing medications. Primary care will need to proactively assess breast cancer risk for women to get the most benefit from risk-based screening and prevention. To promote risk assessment and prevention activities, improved education/training and changes to resources (integrated risk assessment tools, better patient materials etc.) will be necessary.

**Abstract:**

Implementing risk-stratified breast cancer screening is being considered internationally. It has been suggested that primary care will need to take a role in delivering this service, including risk assessment and provision of primary prevention advice. This systematic review aimed to assess the acceptability of these tasks to primary care providers. Five databases were searched up to July–August 2020, yielding 29 eligible studies, of which 27 were narratively synthesised. The review was pre-registered (PROSPERO: CRD42020197676). Primary care providers report frequently collecting breast cancer family history information, but rarely using quantitative tools integrating additional risk factors. Primary care providers reported high levels of discomfort and low confidence with respect to risk-reducing medications although very few reported doubts about the evidence base underpinning their use. Insufficient education/training and perceived discomfort conducting both tasks were notable barriers. Primary care providers are more likely to accept an increased role in breast cancer risk assessment than advising on risk-reducing medications. To realise the benefits of risk-based screening and prevention at a population level, primary care will need to proactively assess breast cancer risk and advise on risk-reducing medications. To facilitate this, adaptations to infrastructure such as integrated tools are necessary in addition to provision of education.

## 1. Introduction

Population based screening programmes aim to detect asymptomatic cancers at an earlier stage to reduce mortality rates and the need for aggressive treatments associated with long term morbidities [1]. A substantial reduction in breast cancer related mortality has been observed since the introduction of mammographic screening programmes [2,3]. However, harms of breast cancer screening include overdiagnosis and false positive test results. Overdiagnosis refers to the diagnosis of breast cancers via screening that would never have caused any clinically apparent symptoms over the course of a person’s lifetime [4]. A false positive result is an abnormality on a screening test that necessitates further investigations, ultimately ruling out the presence of cancer. Whether the benefits of screening outweigh the known harms has been much debated [2,5].

Adopting an alternative risk-based approach to breast cancer screening has the potential to improve the benefit to harm ratio [6]. The development of risk algorithms, such as the Gail and Tyrer–Cuzick models, has made estimation of an individual’s breast cancer risk possible [7,8]. The provision of personalised breast cancer risk estimates would allow screening and prevention services to be offered that are commensurate with the degree of risk, thus improving benefit to harm ratios [9,10]. In the UK, the Predicting Risk of Cancer at Screening (PROCAS) study demonstrated that breast cancer risk information can be collected and communicated to women participating in a population-based mammographic screening programme [11]. International trials are currently ongoing to establish the effectiveness of a risk-based screening regimen in comparison to standard screening practices [12,13].

A key benefit of risk estimation is the ability to identify women at increased risk, affording them the opportunity to benefit from preventative strategies. There are two strategies that have proven benefit in reducing breast cancer risk. The use of selective oestrogen receptor modulators and aromatase inhibitors, commonly referred to as chemoprevention or risk-reducing medication, have been shown to reduce breast cancer incidence [14,15]. Furthermore, evidence suggests 15–40% of breast cancers may be preventable by engaging in health-related behaviours such as increased physical activity and reduced alcohol intake [16]. Clinical guidance acknowledges the need to discuss lifestyle related risk factors in relation to breast cancer risk but the care setting where this discussion should take place is not specified [17].

As the first point of healthcare contact for the general population, primary care has been repeatedly identified as the most opportune setting to conduct breast cancer risk assessment [18,19]. Secondly, primary care providers have a critical role in delivering preventive health care services to the general population as evidenced by their current role in assessment and management of cardiovascular and diabetes risk [20,21].

As the likely roles of primary care in delivering risk-based screening and prevention will be risk assessment and provision of primary prevention advice including prescription of risk-reducing medication, it is important to assess acceptability of these activities. Acceptability is increasingly being recognised as an important component of the feasibility of complex interventions in guidance documents such as the Medical Research Council (MRC) framework [22].

A previous review identified a considerable evidence base related to the acceptability of primary care involvement in risk-based screening and prevention [19]. This review identified numerous barriers reported by primary care providers in relation to their proposed roles which suggests concerns about the acceptability of this approach. However, the scope of the review was limited as it did not examine key participant-reported evaluations of acceptability, such as confidence, as recommended by an evidence-based framework of acceptability [23]. Furthermore, the review did not quantify the strength of individual barriers and facilitators nor examine potential sources of variation such as country and healthcare specialty. The latter is important to investigate as countries vary substantially in how primary healthcare is delivered, including differences in training requirements and what types of providers are considered part of the primary care workforce [18]. Consequently, implementation of risk-based screening and prevention will likely differ across countries [19].

The present systematic review aimed to provide a robust and in-depth examination of acceptability beyond identification of barriers and facilitators. It achieves this by employing the theoretical framework of acceptability which recognises the value of participant-reported evaluations of acceptability in addition to behavioural assessments [23]. However, as primary care providers’ significant knowledge deficits in this area have been described extensively in previous systematic reviews [19,24,25,26], the present review did not assess the extent to which primary care providers understand breast cancer risk assessment and management.

Specific objectives were to summarise the evidence base on:

- ratings of acceptability (including, attitudes, opinions, beliefs, feelings, barriers or facilitators) by primary care providers with respect to (1) breast cancer risk assessment and (2) primary prevention advice
- the performance of routine behaviours by primary care providers regarding (1) breast cancer risk assessment and (2) primary prevention advice
- sources of variation in acceptability and behaviours

## 2. Methods

The protocol of this systematic review was pre-registered in PROSPERO (CRD42020197676) and follows the reporting guidelines detailed in the PRISMA statement [27]. The protocol covered both quantitative and qualitative literature but for reasons of space only the quantitative findings are reported here.

### 2.1. Search Strategy

The following electronic databases were searched: MEDLINE, EMBASE, CINAHL Plus, PsycINFO (each up to 10 July 2020) and ProQuest Dissertations & Theses Global (up to 26 August 2020). Databases were searched from 1989 as the first breast cancer risk model incorporating multiple breast cancer risk factors was published in this year [7]. Search terms were produced using medical subject headings (MeSH), other index terms, keywords and appropriate synonyms (see Supplementary Material S1) and refined with the input of a librarian with expertise in systematic review searching. The strategy was tailored in accordance with the technical language of each database. The searches were limited to articles for which the full text was available in English. Forward and backward citation searches and a lead author search were performed for all included papers. Relevant reviews were hand-searched and researchers with expertise in the area were contacted to identify any additional articles not retrieved by the searches.

### 2.2. Eligibility Criteria

Studies were included in the review if they met the following criteria:

1. Healthcare professionals who provided primary care services. To account for variation in professional roles between healthcare structures in different countries, samples reported as being primary care providers were regarded as such. In ambiguous cases, authors were contacted to clarify whether their samples provided primary care services in line with the World Health Organisation’s definition [28].
  Studies conducted with both primary and secondary care providers were only included if it was possible to separately identify those findings relevant to primary care providers.
2. Data had to be reported about risk assessment and/or providing primary prevention advice in the context of breast cancer. Studies focusing on cancer risk or primary prevention whereby data specific to breast cancer could not be extracted were excluded.
3. Either or both of the following:
  (a) Acceptability defined as anticipated or experiential cognitive and emotional responses. Studies had to report one or more of the following outcomes using quantitative methodologies: attitudes, opinions (e.g., perceptions of responsibility), beliefs, feelings (e.g., confidence), barriers or facilitators.
  (b) Routine behaviours defined as typical or regular activity in clinical practice. Frequency of behaviours reported in a specific timeframe were not eligible for inclusion. Hypothetical clinical scenarios/vignettes or reflections on previous clinical cases were ineligible as these methods ascertain the action taken in a specific situation which may not be indicative of routine behaviours.
4. Studies: Full empirical articles of any quantitative design published in the English language. Grey literature including PhD theses, dissertations and unpublished research were eligible for inclusion. Additionally, baseline surveys of intervention studies designed to improve breast cancer risk assessment behaviours or provision of primary prevention advice were included.

### 2.3. Selection and Coding of Studies

The search results were downloaded into Endnote and duplicates were removed. The library was then uploaded to Rayyan [29] to complete screening. The first author screened all titles and abstracts and a second reviewer (RH) independently screened 30% (k = 945) of these (97% agreement). Full text articles were obtained for all records that appeared to be eligible or could not be confidently excluded (k = 124). The first author read all full text articles and assessed these against the eligibility criteria. A second reviewer (RH) read 50% of the full text articles (k = 62) and disagreements regarding the eligibility of an article were resolved by discussion. In ambiguous cases, additional reviewers were consulted (DF, SH) and consensus was reached.

### 2.4. Data Extraction

Following full text review, detailed information on study characteristics (authors, country, study design and outcome measures), sample characteristics (sample size, age and sex) and outcome data relevant to the objectives were extracted by the first author for all eligible articles. A second reviewer (RH) verified the data extraction by independently extracting primary outcome data for 50% (15/29) of eligible articles.

### 2.5. Quality Assessment

The Mixed Methods Appraisal Tool (MMAT) was deemed most suitable for quality assessment due to its demonstrated reliability and inclusion of quality criteria specifically designed to appraise quantitative descriptive study designs such as surveys [30]. Criteria were categorised as ‘yes’, ‘somewhat’, ‘no’ or ‘can’t tell’. The response option of ‘somewhat’ was added to reflect when a criterion had been partially fulfilled but lacked some key indicators of quality. This enabled a more nuanced approach to quality appraisal. The authors of the tool discourage the use of a scoring metric therefore a narrative description of quality is provided.

In line with MMAT recommendation, two authors (SB and DF) discussed which quality indicators were most important to consider for each criterion listed and following this a coding scheme was devised and agreed upon. All studies were appraised using the criteria for quantitative descriptive designs to assess the quality of the survey design and outcome measures which were of most interest to the review. A 50% rate of response was a priori regarded as satisfactory for avoidance of nonresponse bias, in line with response rates observed in previously published provider surveys [31]. Two authors (SB and RH) independently appraised the quality of the remaining studies. Reviewers met on three separate occasions to check the reliability of decisions and any disagreements were discussed and resolved. During these meetings, the coding scheme was also reviewed and refined in line with discussions to ensure consistency and fairness in coding.

### 2.6. Synthesis of the Evidence

A meta-analysis was deemed inappropriate as studies varied widely in outcomes, measurement scales and study populations. Instead, a narrative synthesis was conducted with findings tabulated [32]. The outcomes from each study were organised into categories initially based on what the authors of each individual study stated the data was measuring (i.e., barrier, facilitator, confidence etc.). Additional outcomes that had not been explicitly measured as barriers or facilitators (e.g., beliefs, feelings, etc.) were reviewed and categorised as such depending on whether they could reasonably be considered to promote or impede performance of the behaviour. For example, a negative affective attitude such as discomfort was categorised as a barrier. Consensus was reached on these decisions through discussion with additional reviewers (DF and SH). Supplementary Material S2 provides full details of the outcomes included per study, the raw data extracted from each study, and how each outcome was categorised.

To aid interpretation and allow meaningful patterns to be identified, outcomes were categorised into broader themes depending on content (see Supplementary Material S2). Initial themes were identified by the first author. These themes were then refined and agreed upon following several rounds of consultation with additional reviewers (DF and SH). The findings were synthesised across the included studies.

## 3. Results

### 3.1. Study Characteristics

**Figure 1:**
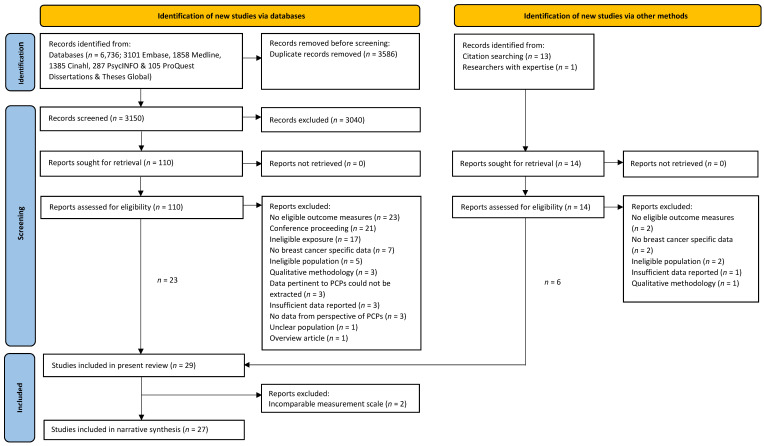
PRISMA flow diagram of study selection.

The searches identified 6750 articles, of which 3164 remained after duplicates were removed (Figure 1). A total of 29 studies were eligible for inclusion (see Supplementary Table S1 for list of excluded studies and reasons). Years of publication ranged from 1997 to 2020. Twenty-seven studies were included in the synthesis. Two were excluded due to using measurement scales that could not be meaningfully compared to other studies [33,34]. More than half of the included studies were conducted in the USA (k = 14) (Table 1). Sample sizes ranged from 28 [35] to 1311 [36] individuals. The most commonly studied population were physicians. The majority (24/27, 89%) of studies assessed at least one outcome relevant to breast cancer risk assessment. In comparison, fewer studies assessed outcomes pertinent to primary prevention (9/27, 33%). No studies investigating health-related behaviours within the context of breast cancer risk were identified so primary prevention findings are limited to risk-reducing medications only.

### 3.2. Perceived Practice Responsibilities with Respect to Both Risk Assessment and Primary Prevention

Primary care providers’ perceptions of responsibility with respect to tasks implicated in breast cancer risk assessment and primary prevention were examined in several studies. Taking a family history was overwhelmingly perceived as a primary care responsibility (88.8–98.1%; Table 2). Additionally, primary care providers readily identified counselling about risk and providing follow up support post genetic testing as practice responsibilities. In comparison, discussion of genetic testing and disclosure of results were less likely to be perceived as primary care responsibilities. Inter-country differences were apparent in a study that recruited participants from four European countries [37]. GPs from France ascribed most practice responsibilities to themselves whereas GPs from the UK considered genetic risk and genetic testing to be the responsibility of genetic specialists. The most commonly assumed responsibilities for primary prevention were writing ongoing prescriptions for risk-reducing medications and initiating discussions about preventative measures.

## 4. Risk Assessment

### 4.1. Barriers and Facilitators

For conducting breast cancer risk assessment, the most commonly endorsed barriers were insufficient education/training followed by discomfort discussing breast density and performing the assessment (Table 3). The least frequently endorsed barriers included lack of primary care responsibility and concern about the implications of risk assessment for women with respect to causing unnecessary anxiety or impacting screening behaviour. None of the included studies investigated factors that could help facilitate breast cancer risk assessment behaviour.

### 4.2. Perceived Confidence

Primary care providers reported highest levels of confidence in taking a family history (60.7–65.5%) and reassuring low-risk patients (46.0–67.7%) (Table 4). Very low levels of confidence were observed for using the Gail model to calculate breast cancer risk (8.6%).

### 4.3. Routine Behaviours

The discussion and collection of breast cancer family history was reported to be a common task (Table 5). Rates were particularly high when the situational context increased the saliency of the topic matter; for example, during a discussion about a woman’s health history or when a woman presented with concerns about breast cancer risk (90.4–92.6%). In comparison, routine collection of family history during a new patient appointment was found to be lower (48.4–69.3%) in two studies conducted in the UK [42,43]. Reported use of multi-factorial risk assessment tools was low with estimates ranging from 3 to 50.9%.

Professional specialty and training level were found to be associated with reported behaviours. A higher proportion of providers specialising in obstetrics and gynaecology reported using risk assessment tools in comparison to family and internal medicine providers [44,45]. Additionally, qualified physicians were significantly more likely to report routinely assessing family history and using the Gail model compared to residents in training [31,44]. 

## 5. Primary Prevention Advice

### 5.1. Barriers and Facilitators

Overall, there was higher endorsement of barriers for primary prevention than risk assessment. The most prevalent barriers for providing primary prevention advice were concern and discomfort prescribing risk-reducing medication and insufficient education/training, in line with barriers to risk assessment (Table 6). Furthermore, a greater proportion of primary care providers were more likely to report they see fewer patients for whom risk-reducing medications are indicated in comparison to patients suitable for risk assessment (39.6% vs. 12.5%). The majority of primary care providers did not report beliefs indicating scepticism about the evidence base underpinning risk-reducing medications. More specifically, few indicated that they believed the risks of prescribing risk-reducing medications outweighed the benefits (6.5–20.5%) or expressed doubts about effectiveness of risk-reducing medications (1.0–31.5%). Lack of scepticism was a consistent finding reported across all five studies that assessed this outcome.

Primary care providers endorsed all facilitators to a relatively high degree (32.0–61.6%; Table 7). Availability of provisions to discuss risk-reducing options more effectively was endorsed as the strongest facilitator for providing primary prevention advice.

Providers specialising in women’s health reported feeling more comfortable using a breast cancer risk assessment tool and prescribing risk-reducing medication [46]. These providers were also less likely to agree that the risks of prescribing risk-reducing medications outweighed the benefits in comparison to other primary care providers [45].

### 5.2. Perceived Confidence and Routine Behaviours

Primary care providers reported low levels of confidence in providing advice/information to patients about risk-reducing medications (24%) [41]. Only one study reported a behavioural outcome relevant to primary prevention wherein 13.5% reported discussing chemoprevention ‘usually’ or ‘always’ [31].

### 5.3. Quality Assessment

Overall, study quality was poor (Table 8). A detailed breakdown of quality assessment by question for each study is available in Supplementary Material S3. For the majority of studies, external validity was likely to be low due to reliance on recruitment through single institutions and sampling via medical association membership lists with limited coverage of the target population. For example, membership of the American Medical Association has been declining with the most recent estimate suggesting only 15% of practising US doctors are members [61]. Inadequate reporting of how outcome measures were developed was common across studies. Furthermore, none of the outcomes of interest were assessed using standardised measures with demonstrated reliability and validity; response rates lower than 50% were reported in k = 14 (48%) studies [34,35,36,37,39,41,45,47,48,50,51,55,58,60].

## 6. Discussion

### 6.1. Summary of Main Findings

The results from this systematic review indicate that primary care providers typically take a reactive role in breast cancer risk assessment that is predominantly focused on collection of family history and provision of support following identification of increased risk. Reported use of multi-factorial risk assessment tools was low. Primary care providers reported higher discomfort and lower confidence with respect to prescribing risk-reducing medications when compared to risk assessment. However, few providers reported beliefs suggestive of doubts about the evidence base underpinning risk-reducing medications. Insufficient education/training and perceived discomfort were amongst the most commonly endorsed barriers reported for both activities. The strongest facilitators for offering risk-reducing medication related to availability of provisions such as clear guidelines and tools to facilitate identification of suitable patients. Professional background, training and country were identified as sources of variation in acceptability and behaviours. The methodological quality of included studies was generally poor and common limitations were high nonresponse rates and use of non-standardised outcome measures.

### 6.2. Relevance to Existing Literature

Previous systematic reviews have consistently identified primary care providers’ lack of knowledge about breast cancer risk assessment and management as a significant barrier to engagement [19,24,25,62]. In line with this, the present review found that insufficient education/training was a prevalent barrier reported for both risk assessment and primary prevention.

Prior to this review, widespread reticence by primary care providers to discuss and prescribe risk-reducing medications has been recognised [60,63,64,65]. In line with previous findings, this review found that primary care providers report high levels of discomfort and low levels of confidence associated with risk-reducing medications. However, the present review also offers novel insight: few primary care providers reported scepticism about the evidence base underpinning risk-reducing medication. This suggests that the perceived discomfort towards risk-reducing medication is not solely attributable to a lack of knowledge. The present findings on facilitators instead highlight that there is a need for more structural approaches, such as the use of guidelines or prompts, to facilitate primary care involvement in breast cancer risk assessment and management practices.

This review has been the first to investigate sources of variation in acceptability and behaviours. Examination of routine behaviours illustrated that primary care providers infrequently report using multi-factorial risk assessments such as the Gail or Tyrer–Cuzick models [7,8]. Nonetheless, family history collection was reported as a common behaviour and perceived as a core task for the majority of primary care providers. Lower levels of routine family history taking were reported by two UK studies [42,43]. However, given the age of these studies (1997 and 2001), these findings may not be reflective of current clinical practice. Nevertheless, present guidelines in the UK and Europe discourage primary care providers from proactively identifying women with a family history of breast cancer [17,37]. A survey of GPs and breast surgeons from four different European countries revealed strong disapproval of the current purely reactive approach to family history assessment [66]. Therefore, the present guidelines are likely to hinder optimal promotion of risk assessment and primary prevention activities in UK and European primary care settings.

Professional background was also associated with outcomes. Providers specialising in women’s health issues reported feeling more comfortable with respect to both risk assessment and primary prevention as evidenced by greater reported use of quantitative risk assessments and fewer negative views about the risks of risk-reducing medications. These perceptions are likely to be the result of specialised knowledge acquired through additional training. For instance, an understanding of the diagnosis and clinical management of hereditary breast and ovarian cancer syndrome is considered essential for obstetrician/gynaecologists [67]. Therefore, in countries such as the USA, primary care providers specialising in women’s health may be more prepared to assume greater responsibility for assessment and management of breast cancer risk because of their knowledge and experience acquired through training and routine practice.

### 6.3. Limitations

The present review has identified methodological biases present in the primary studies. Firstly, high rates of nonresponse were observed in a significant proportion of the studies. Primary care providers who respond to surveys might have more positive views of breast cancer risk assessment and primary prevention than non-responders which may lead to overestimations of acceptability. Furthermore, there was a reliance on convenience sampling procedures in many studies. Outcomes were not assessed using standardised measures with demonstrated reliability and validity. In addition, older studies included in the review may be a poor reflection of current clinical practice given the significant advances made in breast cancer risk assessment and management in recent years. Primary prevention outcomes tended to be assessed in more recent studies which is in line with risk-reducing medications being a relatively new option in comparison to risk assessment. Nonetheless, findings were largely consistent across studies suggesting that methodological limitations with sampling and publication date did not unduly affect the overall conclusions.

Substantial heterogeneity across included studies was evident and therefore a meta-analysis was not possible. To allow meaningful patterns to be identified via narrative synthesis, the research team decided how outcomes were categorised and to some extent this process was subjective. Nevertheless, analytical processes were reviewed in reflective team meetings to achieve consensus and ensure a rigorous and robust synthesis.

Additionally, a wide range of values were observed for some outcomes indicating uncertainty about the average values presented. This is likely to be the result of heterogeneous outcome measurements, as well as differences in samples included. Consequently, caution is warranted when drawing conclusions about the precision of estimating strength of outcomes.

Finally, and perhaps surprisingly, the review did not identify any studies investigating primary care providers’ perceptions of discussing health-related behaviours within the context of breast cancer risk reduction. There is, however, an evidence base focusing on cancer risk more generally. Inclusion of this literature may have provided a more comprehensive understanding of primary care’s perceived role in primary prevention than was possible in this review.

### 6.4. Implications and Future Research Directions

The present review suggests that provision of education/training will be necessary but not sufficient to facilitate primary care involvement in breast cancer risk assessment and primary prevention. The findings on facilitators and routine behaviour indicate that adapting infrastructure and providing prompts to utilise available resources are essential to increase the likelihood of primary care providers routinely conducting both activities. For instance, the integration of risk assessment and management tools into practice software or access to web-based applications would facilitate the desired behaviours, as has been demonstrated for cardiovascular risk assessment and management [20]. Several prototype tools for breast cancer risk assessment have been subject to usability and acceptability testing [68,69]. Primary care providers have expressed concerns about the amount of time needed to complete such tools and highlighted the lack of guidance on clinical management as a significant barrier to use. Therefore, future tool development should focus on streamlining the process and incorporating risk reduction recommendations to increase uptake in routine practice. Additionally, future research should focus on developing and evaluating the impact of educational interventions on knowledge assimilation. This will identify what implementation support primary care will require to fulfil their proposed roles in risk assessment and primary prevention. However, it is worth noting evidence which suggests not all women may be in favour of primary care performing these roles [70]. Therefore, further research assessing the acceptability of this approach to women is needed.

Nursing staff were underrepresented in the included studies. Decision makers have suggested that nurses could assume increased duties in risk assessment and management to support implementation of risk-based screening and prevention [71]. In relation to primary prevention, general practitioners have been found to perceive intervening on obesity as an inappropriate use of their time in comparison to nurses who report feeling responsible for raising the topic [72]. Given the important role of health-related behaviours in reducing breast cancer risk, it would be timely to compare and contrast the views of primary care nurses and physicians to determine their respective roles in implementing prevention recommendations for breast cancer.

Additionally, there is a clear need for more research using populations outside the US to understand the feasibility of primary care assessing and managing breast cancer risk in different healthcare contexts. Future primary studies would benefit from assessing similar outcomes across studies using measures with demonstrated reliability and validity. Wider and more representative sampling frames should be used to obtain better coverage of the target population. Furthermore, recruitment strategies that build personal connections with potential participants such as using physician recruiters ought to be considered to reduce nonresponse rates [73].

## 7. Conclusions

Within the context of implementing risk-based breast cancer screening and prevention, the findings of this review suggest that primary care providers are more likely to accept an increased role in breast cancer risk assessment compared to advising on risk-reducing medications. Adaptations to infrastructure will be necessary to promote enactment of breast cancer risk assessment and management behaviours in addition to provision of education. To fully realise the benefits of risk-based breast cancer screening and prevention, guidelines will need to be reviewed to ensure promotion of a proactive approach to breast cancer risk assessment in primary care.

## Figures and Tables

**Table 1:** Characteristics of studies included in the synthesis (*n* = 27).

Characteristic	Number of Studies
Year of publication	
1997–2004	7
2005–2012	9
2013–2020	11
Study country	
USA	14
UK	5
Switzerland	2
Multiple countries *	2
France	1
Canada	1
Belgium	1
Australia	1
Sample size (*n*)	
1–250	12
251–500	8
501–750	3
751–1000	2
>1000	2
Study population	
Physicians only	16
Mixed ^1^	5
Physicians and nursing staff	4
Nursing staff only	2
Study outcomes	
Risk assessment	24
Primary prevention	9
% women in provider cohort	
0–25	1
26–50	11
51–75	10
76–100	0
Not reported	5

Notes. * Nippert et al. (2014)—France, the Netherlands, UK and Germany; Mainous et al. (2013)—USA and Canada. ^1^ These studies recruited other professional groups in addition to physicians and nursing staff, namely physician assistants, midwives and residents.

**Table 2:** Primary care providers’ perceived responsibilities in breast cancer risk assessment and primary prevention.

Tasks	Percentage Reporting Primary Care Responsibility [Mean and Range Reported if Multiple Values]	Associations with Perceived Roles
Breast cancer risk assessment		
Taking or documenting a family history [31,38,39]	92.7 [89.0–98.0]	
Providing counselling regarding familial risk [38,39]	83.0 [81.0–85.0]	
Providing follow up support after genetic testing [37,38]	79.7 [66.8–92.5]	Country: One study recruited participants from four European countries (UK, France, Germany and the Netherlands). The majority of GPs from all four countries agreed that providing support after breast cancer testing was a primary care responsibility. However, the proportions varied significantly; the highest proportion was reported by the GPs from France (86.1%) and the lowest by the GPs from the UK (57.2%) [37]
Obtaining informed consent before genetic testing [38,39]	77.3 [67.0–87.5]	
Identifying families at risk [38,39]	72.0 [58.0–86.0]	
Calculating breast cancer risk [31]	62.0	
Informing about breast cancer genetic testing [37,39]	61.5 [47.0–76.0]	Country: GPs from France were significantly more likely to assume responsibility for informing patients about breast cancer genetic testing in comparison to GPs from Germany, the Netherlands and the UK (56.2% vs. 46.6%, 41.7% and 41.6%) [37]
Counseling women about breast density [40]	43.0	
Explaining the inheritance pattern of familial breast cancer [37]	42.7	Country: GPs from France were significantly more likely to assume responsibility for explaining the inheritance pattern of familial breast cancer in comparison to GPs from Germany, the Netherlands and the UK (63.6% vs. 30%, 49.7% and 33.8%) [37]
Disclosing breast cancer genetic test results [37,39]	37.2 [27.4–47.0]	Country: GPs from Germany were significantly more likely to assume responsibility for disclosing breast cancer genetic test results in comparison to GPs from France, the Netherlands and the UK (43.7% vs. 23.5%, 11.6% and 16.9%) [37]
Primary prevention		
Writing ongoing prescriptions for risk-reducing medications [41]	97.9	
Providing options for prevention and early detection of breast cancer [38]	86.0	
Initiating discussion of risk-reducing medications [41]	75.0	
Writing first prescription for risk-reducing medications [41]	31.3	
Breast cancer risk reduction with chemopreventive agents [31]	18.0	Sex: Males more likely to agree that breast cancer risk reduction with chemopreventive agents was a primary care provider’s responsibility than females (28% compared to 10%) [31]

**Table 3:** Primary care providers’ perceptions of barriers associated with conducting breast cancer risk assessment.

Themes	Percentage Endorsing Barrier [Mean and Range Reported if Multiple Values]	Associations with Barriers
Insufficient education/training [31,41,44,46]	45.2 [20.0–82.1]	
Discomfort discussing breast density [35,47,48]	36.9 [11.7–81.5]	Training level: Internal medicine providers more likely to agree that they were comfortable counselling women about breast density compared to primary care residents (38% compared to 0%) [35]
Discomfort conducting breast cancer risk assessment [46,49,50]	30.9 [29.3–33.5]	Specialty: Women’s health providers more likely to respond that they were ‘very comfortable/comfortable’ with using a breast cancer risk assessment tool compared to other primary care providers (38% compared to 14%) [46]
More immediate issues to discuss during consultation [31]	25.0	
Insufficient provisions to conduct breast cancer risk assessment effectively (e.g., tools, patient information etc.) [31,41,44,46,51]	20.6 [11.0–40.0]	
Perceived lack of impact on patient management [44,46]	16.8 [7.9–25.6]	
Low perceived utility and acceptability of genetic testing for determining breast cancer risk [36,52]	14.0 [5.1–22.9]	
Concern that risk prediction models are not accurate enough [51]	13.0	
Do not see patients for whom risk assessment is indicated [44,46]	12.5 [7.9–17]	
Concern about creating unnecessary anxiety/worry for many women [51]	7.9 [2.0–13.7]	
Assessment of breast cancer risk is not part of routine practice [41]	7.0	
Perceived lack of primary care responsibility [46]	5.9	
Reluctance to assess risk because a woman at low risk of breast cancer might decide not to undergo mammography screening [51]	6.0	

**Table 4:** Primary care providers’ perceived confidence in performing breast cancer risk assessment behaviours.

Tasks	Percentage Reporting Confidence [Mean and Range Reported if Multiple Values]	Associations with Confidence
Taking a family history [42,53,54]	63.5 [60.7–65.5]	Training: Nurses who had attended training about genetic issues in the 12 months were more likely to report being ‘confident or very confident’ compared with those who did not attend (72% compared to 59%) [42]
Reassuring low-risk patients [42,53,54]	58.8 [46.0–67.7]	Training: Confidence providing reassurance for those at low risk of breast cancer was significantly associated with attending training about genetic issues [42]
Making a basic risk assessment [42,53]	57.4 [53.9–60.8]	
Ability to provide information to patients about *BRCA* cancer risks and inheritance [55]	55.8 [50.0–61.6]	
Ability to provide information to patients about *BRCA* test methods and interpretation [55]	39.6 [37.2–41.9]	
Ability to answer patients’ questions during a consultation about risk [54]	23.2	
Ability to use Gail scores to identify women at increased risk for breast cancer [31]	8.6	

**Table 5:** Primary care providers’ reported behaviours with respect to breast cancer risk assessment.

Behaviours	Percentage Reporting Behaviour [Mean and Range Reported if Multiple Values]	Associations with Behaviour
Breast cancer risk assessment		
Discussing family history as part of a woman’s health history [56]	92.6	
Considering a discussion of family history with a woman consulting with concerns about breast cancer risk [57]	90.4	
Collecting family history during routine clinical practice [31,46,58]	86.3 [71.0–95.0]	Training level: Staff more likely to report ‘usually or always’ assessing family history during routine visits compared to residents (79% compared to 58%) [31]
Discussing family history to assess breast cancer risk [45,49]	67.0 [37.1–96.9]	
Collecting family history during new patient appointment [42,43]	58.9 [48.4–69.3]	
Using multi-factorial breast cancer risk assessment tools [45,47,50]	33.1 [22.4–50.9]	Specialty: Obstetric-gynaecologists more likely to report using breast cancer risk assessment tools compared to family medicine physicians and internists to (67.2% vs. 44.0% and 41.7%) [45]
Assessing risk using the Gail model [31,44,49]	16.8 [3.0–40.9]	Training level: Attending physicians more likely to report use of the Gail model compared to resident physicians [44] Specialty: Gynaecology more likely to report use of the Gail model compared to family medicine and internal medicine physicians (60% vs. 33.3% and 36.9%) [44]

**Table 6:** Primary care providers’ perceptions of barriers associated with providing primary prevention advice.

Themes	Percentage Endorsing Barrier [Mean and Range Reported if Multiple Values]	Associations with Barriers
Discomfort prescribing risk-reducing medication [44,46]	75.0 [70.1–79.8]	Specialty: Women’s health providers more likely to respond that they were ‘very comfortable/comfortable’ with prescribing risk-reducing medication compared to other primary care providers (9% compared to 2%) [46]
Concern about prescribing off-label (unlicensed) medication [50]	58.1	
Never seen a patient for whom risk-reducing medications are indicated [44,46]	39.6 [18.4–60.7]	
Insufficient education/training [41,46,50,59]	34.6 [13.9–72.0]	
Insufficient provisions to discuss risk-reducing measures effectively (e.g., time, patient information, resources etc.) [41,44,46,50,59,60]	22.7 [6.1–50]	Specialty: Family and internal medicine physicians more likely to report time constraints as a barrier than obstetrician-gynaecologists (45.8% and 46.5% vs. 31.3%, respectively) [59]
More immediate issues to discuss during consultation [41]	18.0	
Doubts about effectiveness of risk-reducing medications (e.g., belief in ability to reduce risk and mortality, perceiving the evidence base as controversial) [41,44,45,50,60]	15.4 [1.0–31.5]	
Forgetting to discuss risk-reducing medications [41]	14.0	
Believing that the risks of prescribing risk-reducing medications outweigh the benefits [45,50,60]	13.5 [6.5–20.5]	Specialty: Obstetrician-gynaecologists less likely to agree that the evidence of preventive agents reducing breast cancer risk is controversial compared to family medicine physicians and internists (22.8% vs. 37.6% and 34.0% respectively) [45] Obstetrician-gynaecologists less likely to agree that the risk of endometrial cancer is too great to prescribe tamoxifen for breast cancer reduction compared to family medicine physicians and internists (14.8% vs. 18.4% and 18.8%) [45]. Obstetrician-gynaecologists less likely to agree that the risk of thromboembolic disease is too great to prescribe preventive agents for breast cancer reduction compared to family medicine physicians and internists (10.8% vs. 26.0% and 24.8%) [45]
Women’s perceived lack of interest and knowledge about risk reduction [41,59]	12.0 [1.0–27.0]	
Perceived lack of primary care responsibility [41,46,59]	11.6 [4.0–23.9]	
Lack of incentives for discussing risk reducing measures [41,59]	8.3 [3.0–13.6]	
Discomfort prescribing a ‘cancer drug’ to healthy women [41]	4.0	
Concern about increasing patient’s worry about breast cancer [41]	2.0	
Perceived lack of impact on patient management [46]	1.2	

**Table 7:** Primary care providers’ perceptions of facilitators associated with providing primary prevention advice.

Themes	Percentage Endorsing Facilitator [Mean and Range Reported if Multiple Values]
Availability of provisions to discuss risk-reducing options more effectively (e.g., tools and guidelines to identify suitable patients, better patient education materials etc.) [41,59]	61.6 [33.0–88.0]
Knowing some risk-reducing medications are available at a Government-subsidised price [41]	54.0
Endorsement as part of role by a professional body [41]	53.0
More education/training [59]	52.0 [34.5–69.4]
Patient has indications of increased breast cancer risk [41]	46.3 [36.0–54.0]
Understanding the benefits of primary prevention [41,59]	44.0 [14.0–59.1]
Peer support [41]	41.7 [27.0–64.0]
Believing that the benefits of preventive agents in breast cancer outweigh the risks [45,50]	37.6 [12.4–62.8]
Easier to discuss risk-reducing medications than bilateral mastectomy [41]	32.0

**Table 8:** Quality assessment results for studies included in the review (*n* = 29).

	Yes		Somewhat		No		Cannot Tell	
	*n*	%	*n*	%	*n*	%	*n*	%
Is the sampling strategy relevant to address the research question?	6	21	17	59	3	10	3	10
Is the sample representative of the target population?	9	31	5	17	7	24	8	28
Are the measurements appropriate?	0	0	18	62	0	0	11	38
Is the risk of nonresponse bias low?	3	10	11	38	12	41	3	10
Is the statistical analysis appropriate to answer the research question?	23	79	2	7	0	0	4	14

## Data Availability

The data presented in this review are available in Supplementary Material S2.

## Supplementary Materials

The following are available online at https://www.mdpi.com/article/10.3390/cancers13164150/s1, Material S1: Search terms for each database. Material S2: Data extracted from all primary studies, showing how each eligible outcome mapped onto broader themes reported in the main analyses. Material S3: Summary and detailed results of the quality appraisal using the Mixed Methods Appraisal Tool. Table S1: A list of 95 excluded studies and reasons for exclusion.
