# Supplementary material for: Breast Cancer Risk Assessment and Primary Prevention Advice in Primary Care: A Systematic Review of Provider Attitudes and Routine Behaviours
Source: Cancers (Basel). 2021 Aug 18;13(16):4150. doi: 10.3390/cancers13164150 (PMC8394615; doi:10.3390/cancers13164150)
Supplement: Supplementary file 1 [file cancers-13-04150-s001.zip › cancers-1340598-Supplementary material S1.pdf]

**Material S1:** Search terms for each database

**MEDLINE**

#1

acceptab* OR perception* OR perceiv* OR perspective* OR opinion* OR attitude* OR view* OR interest* OR belie* OR knowledge OR practice* OR experience* OR behavio*

#2

((general or family) ADJ3 (practitioner* or physician* or doctor* or clinician*)) OR ((nurs*) ADJ3 (practitioner* OR specialist* OR staff OR personnel OR clinician*) OR "primary care" OR "general practice" OR exp Primary Health Care/ [MESH]

#3

breast AND (cancer OR neoplasm OR carcinoma OR tumor* OR tumour*)) OR exp Breast Neoplasms/ [MESH]

#4

risk assess* OR (assess* ADJ2 risk)) OR exp Risk Assessment/ [MESH]

#5

(prevent* OR chemoprevent* OR tamoxifen OR raloxifene OR anastrazole) OR exp Primary Prevention/ [MESH]

#6

(lifestyle OR diet* OR smok* OR tobacco OR nicotine OR alcohol OR weight OR activ* OR behavio*) OR (health* ADJ1 behavio*)

#1 AND #2 AND #3 AND #4 **+** #1 AND #2 AND #3 AND #5 **+** #1 AND #2 AND #3 AND #6

Limits applied to each combination: 1) publication date from 01.01.1989-10.07.2020 & 2) English language

**Embase**

#1
acceptab* OR perception* OR perceiv* OR perspective* OR opinion* OR attitude* OR view* OR interest* OR belie* OR knowledge OR practice* OR experience* OR behavio*

#2

((general or family) ADJ3 (practitioner* or physician* or doctor* or clinician*)) OR ((nurs*) ADJ3 (practitioner* OR specialist* OR staff OR personnel OR clinician*) OR "primary care" OR "general practice" OR exp Primary Health Care/ [MESH]

#3

breast AND (cancer OR neoplasm OR carcinoma OR tumor* OR tumour*)) OR exp Breast Tumor/ [MESH]

#4

risk assess* OR (assess* ADJ2 risk)) OR exp Risk Assessment/ [MESH]

#5

(prevent* OR chemoprevent* OR tamoxifen OR raloxifene OR anastrazole) OR exp Primary Prevention/ [MESH] OR exp Preventive Medicine/ [MESH]

#6

(lifestyle OR diet* OR smok* OR tobacco OR nicotine OR alcohol OR weight OR activ* OR behavio*) OR (health* ADJ1 behavio*)

#1 AND #2 AND #3 AND #4 **+** #1 AND #2 AND #3 AND #5 **+** #1 AND #2 AND #3 AND #6

Limits applied to each combination: 1) publication date from 01.01.1989-10.07.2020 & 2) English language

**CINAHL Plus**

#1
acceptab* OR perception* OR perceiv* OR perspective* OR opinion* OR attitude* OR view* OR interest* OR belie* OR knowledge OR practice* OR experience* OR behavio*

#2

((general or family) N3 (practitioner* or physician* or doctor* or clinician*)) OR ((nurs*) N3 (practitioner* OR specialist* OR staff OR personnel OR clinician*) OR "primary care" OR "general practice" OR MM "Primary Health Care"

#3

breast AND (cancer OR neoplasm OR carcinoma OR tumor* OR tumour*)) OR "Breast Neoplasms+" [MESH]

#4

(risk assess* OR (assess* N2 risk)) OR MM "Risk Assessment"

#5

(prevent* OR chemoprevent* OR tamoxifen OR raloxifene OR anastrazole) OR "Preventive Health Care+" [MESH]

#6

(lifestyle OR diet* OR smok* OR tobacco OR nicotine OR alcohol OR weight OR activ* OR behavio*) OR (health* N1 behavio*)

#1 AND #2 AND #3 AND #4 **+** #1 AND #2 AND #3 AND #5 **+** #1 AND #2 AND #3 AND #6

Limits applied to each combination: 1) publication date from 01.01.1989-10.07.2020 & 2) English language

**PsycINFO**

#1

acceptab* OR perception* OR perceiv* OR perspective* OR opinion* OR attitude* OR view* OR interest* OR belie* OR knowledge OR practice* OR experience* OR behavio*

#2

((general or family) ADJ3 (practitioner* or physician* or doctor* or clinician*)) OR ((nurs*) ADJ3 (practitioner* OR specialist* OR staff OR personnel OR clinician*) OR “primary care” OR “general practice” OR exp Primary Health Care/ [MESH]

#3

breast AND (cancer OR neoplasm OR carcinoma OR tumor* OR tumour*)) OR exp Breast Neoplasms/ [MESH]

#4

risk assess* OR (assess* ADJ2 risk)) OR exp Risk Assessment/ [MESH]

#5

(prevent* OR chemoprevent* OR tamoxifen OR raloxifene OR anastrazole) OR exp preventive medicine/ [MESH]

#6

(lifestyle OR diet* OR smok* OR tobacco OR nicotine OR alcohol OR weight OR activ* OR behavio*) OR (health* ADJ1 behavio*)

#1 AND #2 AND #3 AND #4 **+** #1 AND #2 AND #3 AND #5 **+** #1 AND #2 AND #3 AND #6

Limits applied to each combination: 1) publication date from 01.01.1989-10.07.2020 & 2) English language

**ProQuest Dissertations & Theses Global**

#1

accept* OR perception* OR perceiv* OR perspective* OR opinion* OR attitude* OR view* OR interest* OR belie* OR practice* OR knowledge OR experience* OR behavio*

#2

((general OR family) NEAR/3 (practitioner* OR physician* OR doctor* OR clinician*)) OR nurs* NEAR/3 (practitioner* OR specialist* OR staff OR personnel OR clinician*) OR "primary care" OR "general practice"

#3

breast AND (cancer OR neoplasm OR carcinoma OR tumor* OR tumour*)

#4

(risk assess*) OR (assess* NEAR/2 risk)

#5

(prevent* OR chemoprevent* OR tamoxifen OR raloxifene OR anastrazole)

#6

(lifestyle OR diet* OR smok* OR tobacco OR nicotine OR alcohol OR weight OR activ* OR behavio*) OR (health* NEAR/1 behavio*)

#1 AND #2 AND #3 AND #4 **+** #1 AND #2 AND #3 AND #5 **+** #1 AND #2 AND #3 AND #6

Limits applied to each combination: 1) publication date from 01.01.1989-26.08.2020 & 2) English language
