# Supplementary material for: Breast Cancer Risk Assessment and Primary Prevention Advice in Primary Care: A Systematic Review of Provider Attitudes and Routine Behaviours
Source: Cancers (Basel). 2021 Aug 18;13(16):4150. doi: 10.3390/cancers13164150 (PMC8394615; doi:10.3390/cancers13164150)
Supplement: Supplementary file 1 [file cancers-13-04150-s001.zip › cancers-1340598-Supplementary material S2.pdf]

Material S2. Data extracted from all primary studies, showing how each eligible outcome mapped onto broader themes reported in the main analyses

**Primary care providers' perceived responsibilities in breast cancer risk assessment and primary prevention: data extracted from primary studies for Table 2, mapping onto responses reported in main analyses**				
**Reference (author & year)**	**Eligible outcomes**	**Theme**	**Eligible response scale option(s)**	**Raw data extracted (%)**
Escher & Sappino (2000)	Document a family history of cancer	Taking or documenting a family history	Family physician	89.0
	Recognise families for which genetic testing is indicated	Identifying families at risk	Family physician	86.0
	Provide counselling regarding familial risk	Providing counselling regarding familial risk	Family physician	81.0
	Obtain informed consent before testing	Obtaining informed consent before genetic testing	Family physician	87.5
	Provide follow up support	Providing follow up support after genetic testing	Family physician	92.5
	Provide options for prevention and early detection of breast cancer	Providing options for prevention and early detection of breast cancer	Family physician	86.0
Gunn et al. (2018)	Counselling women about breast density is primarily my responsibility as a primary care physician versus the responsibility of other clinicians or imaging providers	Counselling women about breast density	Strongly agree or agree	43.0
Macdonald et al. (2020)	Initiate discussion of risk-reducing medications	Initiating discussion of risk-reducing medications	GP	75.0
	Write first prescription	Writing first prescription for risk-reducing medications	GP	31.3
	Write ongoing prescriptions	Writing ongoing prescriptions for risk-reducing medications	GP	97.9
Nippert et al. (2014)	Explain the inheritance pattern of familial breast cancer	Explaining the inheritance pattern of familial breast cancer	Myself (GP)	42.7*
	Inform about breast cancer genetic testing	Informing about breast cancer genetic testing	Myself (GP)	47.0*
	Disclose the breast cancer genetic test results to the patient	Disclosing breast cancer genetic test results	Myself (GP)	27.4*
	Provide support after breast cancer testing	Providing follow up support after genetic testing	Myself (GP)	66.8*
Pichert et al. (2003)	Do you think it is your duty to inform an individual at high risk for breast cancer that genetic counselling and testing is available?	Informing about breast cancer genetic testing	Yes	76.0
	Document family history	Taking or documenting a family history	Yes	91.0

	Identify families at risk	Identifying families at risk	Yes	58.0
	Perform genetic counselling	Providing counselling regarding familial risk	Yes	85.0
	Obtain informed consent	Obtaining informed consent before genetic testing	Yes	67.0
	Who should disclose genetic test results?	Disclosing breast cancer genetic test results	Primary care physician	47.0
Sabatino et al. (2007)	Taking a family history	Taking or documenting a family history	Definitely or mostly agree	98.0
	Calculating breast cancer risk	Calculating breast cancer risk	Definitely or mostly agree	62.0
	Breast cancer risk reduction with chemopreventive agents	Breast cancer risk reduction with chemopreventive agents	Definitely or mostly agree	18.0

Notes.
*Percentage manually calculated for whole sample (countries combined)

Primary care providers' perceptions of barriers associated with conducting breast cancer risk assessment: data extracted from primary studies for Table 3, mapping onto responses reported in main analyses				
**Reference (author & year)**	**Eligible outcomes**	**Theme**	**Eligible response scale option(s)**	**Raw data extracted (%)**
Bidassie et al. (2020)	Not enough time	Insufficient provisions to conduct breast cancer risk assessment effectively	N/A – reported by participant as free text	15.8
	Not familiar enough with it	Insufficient education/training	N/A – reported by participant as free text	50.5
	Never seen a patient for whom it was indicated	Do not see patients for whom risk assessment is indicated	N/A – reported by participant as free text	5.9
	Don't think it will impact patient management	Perceived lack of impact on patient management	N/A – reported by participant as free text	7.9
	Not a primary care responsibility	Perceived lack of primary care responsibility	N/A – reported by participant as free text	5.9
	How comfortable are you using the Gail (or other) risk model to assess a woman's risk of breast cancer?	Discomfort conducting breast cancer risk assessment	Very uncomfortable or uncomfortable	33.5
Casas et al. (2017)	I am comfortable counselling women about breast density	Discomfort discussing breast density	Strongly disagree or disagree or neutral	81.5*
Corbelli et al. (2014)	I do not see patients in whom calculation of the Gail score is indicated	Do not see patients for whom risk assessment is indicated	Strongly agree or somewhat agree	17.0
	I do not have enough time with my patients to use the Gail score	Insufficient provisions to conduct breast cancer risk assessment effectively	Strongly agree or somewhat agree	40.0
	I do not think that the results of the Gail score would change my management	Perceived lack of impact on patient management	Strongly agree or somewhat agree	25.6
	I am not sufficiently familiar with the Gail score	Insufficient education/training	Strongly agree or somewhat agree	82.1
Edwards et al. (2009)	Perceived comfort level of breast cancer risk assessment	Discomfort conducting breast cancer risk assessment	Not comfortable	29.3[1]
Guerra et al. (2009)	Information about risk creates unnecessary anxiety for many women	Concern about creating unnecessary anxiety for many women	Strongly agree or agree	13.7
	Available methods of predicting risk are not accurate enough	Concern that risk prediction models are not accurate enough	Strongly agree or agree	13.1
	Too time consuming to evaluate and discuss risk	Insufficient provisions to conduct breast cancer risk assessment effectively	Strongly agree or agree	10.5
	Reluctant to use breast cancer risk assessment because a woman at low risk of breast cancer might decide not to undergo mammography screening	Reluctance to assess risk because a woman at low risk of breast cancer might decide not to undergo mammography screening	Strongly agree or agree	6.3
Khong et al. (2015)	How comfortable do you feel answering questions about breast density?	Discomfort discussing breast density	Not comfortable	11.7

Macdonald et al. (2020)	I have inadequate training and confidence in BC risk assessment	Insufficient education/training	Strong barrier	28.0
	I find it hard to access resources to help me estimate patients' risk	Insufficient provisions to conduct breast cancer risk assessment effectively	Strong barrier	20.0
	I don't routinely assess BC risk with my patients	Assessment of breast cancer risk is not part of routine practice	Strong barrier	7.0
Maimone et al. (2017)	How comfortable are you (or would you be) in answering questions regarding breast density and offering appropriate management recommendations?	Discomfort discussing breast density	Not comfortable	17.1
Mainous et al. (2013)	Respondents asked to estimate the utility of current genetic testing capabilities in determining a patient's risk for breast cancer	Low perceived utility and acceptability of genetic testing for determining breast cancer risk	Not useful	5.1
Sabatino et al. (2007)	Too many things to do during visits	Insufficient provisions to conduct breast cancer risk assessment effectively	Most important	19.0
	Lack of confidence in one's knowledge of risk and risk assessment	Insufficient education/training	Most important	20.0
	More immediate issues	More immediate issues to discuss during consultation	Most important	25.0
Tighe (2009)	Are you comfortable estimating a woman's individual risk for breast cancer?	Discomfort conducting breast cancer risk assessment	No	30.0
Welkenhuysen & Evers-Kiebooms et al. (2002)	The acceptability (how sensible, meaningful or relevant) of performing a predictive test for an asymptomatic adult with a family history of breast cancer	Low perceived utility and acceptability of genetic testing for determining breast cancer risk	Not at all acceptable or not acceptable or somewhat not acceptable	22.9[2]

Notes.
*****Manually calculated for whole sample (residents & providers combined)
[1]Manually calculated using frequencies reported in Figure 2
[2]Manually calculated from percentages provided by the senior author as incorrect n reported in paper

Primary care providers' perceived confidence in performing breast cancer risk assessment behaviours: data extracted from primary studies for Table 4, mapping onto responses reported in main analyses				
**Reference (author & year)**	**Eligible outcomes**	**Theme**	**Eligible response scale option(s)**	**Raw data extracted (%)**
Bankhead et al. (2001)	Knowing the relevant family history to take	Taking a family history	Very confident or confident	60.7
	Making a basic risk assessment	Making a basic risk assessment	Very confident or confident	60.8
	Reassuring women at low risk	Reassuring low-risk patients	Very confident or confident	62.6
Bethea et al. (2008)	Knowing the relevant family history to take	Taking a family history	Very confident or confident	65.5
	Making a basic assessment of level of risk	Making a basic risk assessment	Very confident or confident	53.9
	Reassuring women at low risk	Reassuring low-risk patients	Very confident or confident	67.7
Dekanek et al. (2020)	BRCA cancer risks	Ability to provide information to patients about BRCA cancer risks and inheritance	Completely or somewhat confident	61.6
	BRCA inheritance	Ability to provide information to patients about BRCA cancer risks and inheritance	Completely or somewhat confident	50.0
	BRCA testing methods	Ability to provide information to patients about BRCA test methods and interpretation	Completely or somewhat confident	37.2
	BRCA test interpretation	Ability to provide information to patients about BRCA test methods and interpretation	Completely or somewhat confident	41.9
Sabatino et al. (2007)	Confidence in ability to use Gail scores to identify women at increased risk for breast cancer	Ability to use Gail scores to identify women at increased risk for breast cancer	Very confident or confident	8.6
Wilson et al. (2006)	Taking appropriate family history	Taking a family history	Very or moderately confident	64.3*
	Reassuring low-risk patients	Reassuring low-risk patients	Very or moderately confident	46.0*
	Being able to answer questions	Ability to answer patients' questions during a consultation about risk	Very or moderately confident	23.2*

Notes.
*Manually calculated for whole sample (baseline intervention and control groups combined)

Primary care providers' reported behaviours with respect to breast cancer risk assessment: data extracted from primary studies for Table 5, mapping onto responses reported in main analyses				
**Reference (author & year)**	**Eligible outcomes**	**Theme**	**Eligible response scale option(s)**	**Raw data extracted (%)**
Bankhead et al. (2001)	Routine collection of family history for breast cancer at new patient appointments	Collecting family history during new patient appointment	Yes	69.3
Bidassie et al. (2020)	Do you routinely gather a woman's family history of breast cancer?	Collecting family history during routine clinical practice	Yes	92.8
Corbelli et al. (2014)	Do you calculate the Gail score as part of your clinical practice?	Assessing risk using the Gail model	Yes (even if rarely)	40.9
Edwards et al. (2009)	Using family history as a tool for assessing patient's risk for breast cancer	Discussing family history to assess breast cancer risk	Yes	37.1
	Using the Gail model as a tool for assessing patient's risk for breast cancer	Assessing risk using the Gail model	Yes	6.5
Ganry & Boche (2005)	Systematically looked for a family history of breast cancer	Collecting family history during routine clinical practice	Yes	95.0
Hall (2001)	As part of a woman's health history, do you inquire about her breast cancer family history?	Discussing family history as part of a woman's health history	Always	92.6
Khong et al. (2015)	Do you perform quantitative breast cancer risk assessments (i.e. assessments using Gail, Claus, BRCAPRO, or other standard risk factors) in your office?	Using multi-factorial breast cancer risk assessment tools	Yes	26.0
Sabatino et al. (2007)	The frequency with which providers assess family history during routine visits	Collecting family history during routine clinical practice	Always or usually	71.0
	The frequency with which providers assess risk by Gail score during routine visits	Assessing risk using the Gail model	Always or usually	3.0
Samimi et al. (2020)	How often do you discuss cancer family history in an asymptomatic patient to determine risk for breast and/or ovarian cancer?	Discussing family history to assess breast cancer risk	Used	96.9
	How often do you use a breast cancer risk assessment tool in an asymptomatic patient to determine risk for breast and/or ovarian cancer?	Using multi-factorial breast cancer risk assessment tools	Used	50.9
Summerton & Garrood (1997)	Routine family history enquiries: whether the history of breast cancer in siblings or parents was specifically enquired about on first registration	Collecting family history during new patient appointment	Yes	48.4
Tighe (2009)	If you were concerned that a woman might be at high risk for breast cancer, what would you do? Estimate her risk using available risk assessment models	Using multi-factorial breast cancer risk assessment tools	Yes	22.4

Walter et al. (2001)	Considering discussing family history with a woman consulting with concerns about her risk of breast cancer	Considering a discussion of family history with a woman consulting with concerns about breast cancer risk	Yes	90.4*

Notes.
*Manually calculated for whole sample (general practitioners & nurses combined)

Primary care providers' perceptions of barriers associated with providing primary prevention advice: data extracted from primary studies for Table 6, mapping onto responses reported in main analyses				
**Reference (author & year)**	**Eligible outcomes**	**Theme**	**Eligible response scale option(s)**	**Raw data extracted (%)**
Armstrong et al. (2006)	The risk of endometrial cancer is too great to prescribe tamoxifen for breast cancer risk reduction	Believing that the risks of prescribing risk-reducing medications outweigh the benefits	Strongly agree or agree	15.0
	The evidence that tamoxifen significantly reduces breast cancer risk is controversial	Doubts about effectiveness of risk-reducing medications	Strongly agree or agree	27.7
	It is too time consuming to discuss taking tamoxifen with women in my practice	Insufficient provisions to discuss risk-reducing measures effectively	Strongly agree or agree	19.3
	The risk of thromboembolic disease is too great to prescribe tamoxifen for breast cancer risk reduction	Believing that the risks of prescribing risk-reducing medications outweigh the benefits	Strongly agree or agree	12.3
Bidassie et al. (2020)	Not familiar enough with it	Insufficient education/training	N/A – reported by participant as free text	54.0
	Not a primary care responsibility	Perceived lack of primary care responsibility	N/A – reported by participant as free text	23.9
	Never seen a patient for whom it was indicated	Never seen a patient for whom risk-reducing medications are indicated	N/A – reported by participant as free text	18.4
	Not enough time	Insufficient provisions to discuss risk-reducing measures effectively	N/A – reported by participant as free text	6.1
	Don't think it will impact patient management	Perceived lack of impact on patient management	N/A – reported by participant as free text	1.2
	How comfortable are you at prescribing medication for primary prevention of breast cancer (ie, tamoxifen, raloxifene, exemestane)?	Discomfort prescribing risk-reducing medication	Very uncomfortable or uncomfortable	70.1
Corbelli et al. (2014)	I do not believe that chemoprevention benefits most women who are eligible to receive it	Doubts about effectiveness of risk-reducing medications	Strongly agree or somewhat agree	10.7
	I am not comfortable prescribing chemoprevention	Discomfort prescribing risk-reducing medication	Strongly agree or somewhat agree	79.8
	I do not have time to discuss chemoprevention with my patients	Lack of provisions to discuss risk-reducing measures effectively	Strongly agree or somewhat agree	32.1
	I have not identified a patient in whom chemoprevention was indicated	Never seen a patient for whom risk-reducing medications are indicated	Strongly agree or somewhat agree	60.7
Kaplan et al. (2005)	Not sufficiently informed about risk reduction options	Insufficient education/training	Very often or fairly often	19.1
	Not sufficiently trained in counselling	Insufficient education/training	Very often or fairly often	13.9

	Not my role/should be done by someone else	Perceived lack of primary care responsibility	Very often or fairly often	6.8
	Lack of patient interest	Women's perceived lack of interest and knowledge about risk reduction	Very often or fairly often	12.6
	Patient would not understand	Women's perceived lack of interest and knowledge about risk reduction	Very often or fairly often	7.2
	Not enough time	Insufficient provisions to discuss risk-reducing measures effectively	Very often or fairly often	40.3
	Reimbursement not sufficient	Lack of incentives for discussing risk reducing measures	Very often or fairly often	13.6
Macdonald et al. (2020)	I have insufficient knowledge of risk-reducing medications	Insufficient education/training	Strong barrier	49.0
	I am not confident in providing advice to patients about risk-reducing medications	Insufficient education/training	Strong barrier	32.0
	I have difficulty identifying patients suitable for risk-reducing medications	Insufficient provisions to discuss risk-reducing measures effectively	Strong barrier	29.0
	There are no procedures that encourage me to discuss risk-reducing medications	Insufficient provisions to discuss risk-reducing measures effectively	Strong barrier	24.0
	I have difficulty explaining the pros and cons of risk-reducing medications	Insufficient education/training	Strong barrier	23.0
	I find it hard to access good information for my patients	Insufficient provisions to discuss risk-reducing measures effectively	Strong barrier	21.0
	Medication side effects	Concern about medication side effects	Strong barrier	14.0
	I forget to discuss risk-reducing medications with my patients	Forgetting to discuss risk-reducing medications	Strong barrier	14.0
	Lack of time during consultation	Insufficient provisions to discuss risk-reducing measures effectively	Strong barrier	10.0
	It is difficult to measure whether the medication is working	Insufficient provisions to discuss risk-reducing measures effectively	Strong barrier	7.0
	It is not my role to discuss risk-reducing medications	Perceived lack of primary care responsibility	Strong barrier	4.0
	I feel uncomfortable prescribing a 'cancer drug' to healthy women	Discomfort prescribing a 'cancer drug' to healthy women	Strong barrier	4.0
	There is no evidence that they reduce mortality	Doubts about effectiveness of risk-reducing medications	Strong barrier	4.0

	There are no incentives for discussing risk-reducing medications with patients	Lack of incentives for discussing risk reducing measures	Strong barrier	3.0
	I'm concerned I might increase the patient's worry about breast cancer	Concern about increasing patient's worry about breast cancer	Strong barrier	2.0
	I don't think patients want to discuss risk-reducing medications for cancer prevention	Women's perceived lack of interest and knowledge about risk reduction	Strong barrier	1.0
	I don't believe they decrease the risk of breast cancer	Doubts about effectiveness of risk-reducing medications	Strong barrier	1.0
	There are other things I wish to achieve in most consultations	More immediate issues to discuss during consultation	Strong barrier	18.0
Samimi et al. (2020)	The benefits of preventive agents in breast cancer outweigh the risks	Believing that the risks of prescribing risk-reducing medications outweigh the benefits	Disagree	9.1
	The evidence that preventive agents significantly reduces breast cancer risk is controversial	Doubts about effectiveness of risk-reducing medications	Agree	31.5
	The risk of endometrial cancer is too great to prescribe tamoxifen for breast cancer reduction	Believing that the risks of prescribing risk-reducing medications outweigh the benefits	Agree	17.3
	The risk of thromboembolic disease is too great to prescribe preventive agents for breast cancer reduction	Believing that the risks of prescribing risk-reducing medications outweigh the benefits	Agree	20.5
	It is easy for me to determine who is eligible to take preventive agents for breast cancer reduction	Difficulty identifying patients who are eligible for risk-reducing medications	Disagree	50.0
Tighe et al. (2009)	Not familiar with chemoprevention strategies	Insufficient education/training	N/A – selected outcome from pre-specified list	72.0
	Not enough resources available	Insufficient provisions to discuss risk-reducing measures effectively	N/A – selected outcome from pre-specified list	16.0
	Do you find it too time consuming to assess breast cancer risk and discuss breast cancer prevention strategies with women in your practice?	Insufficient provisions to discuss risk-reducing measures effectively	Yes	17.9
	Do you have concerns about prescribing chemopreventive agents for off label reasons such as primary prevention of breast cancer?	Concern about prescribing off-label (unlicensed) medication	Yes	58.1
	Do you think the evidence showing that chemoprevention significantly reduces breast cancer is controversial?	Doubts about effectiveness of risk-reducing medications	Yes	17.3

	Do you believe that the benefits of chemoprevention for breast cancer outweigh the risks?	Believing that the risks of prescribing risk-reducing medications outweigh the benefits	No	6.5
	How difficult is it to determine who is eligible to take chemopreventive agents for breast cancer risk reduction?	Difficulty identifying patients who are eligible for risk-reducing medications	Difficult	22.2

Primary care providers' perceptions of facilitators associated with providing primary prevention advice: data extracted from primary studies for Table 7, mapping onto responses reported in main analyses				
**Reference (author & year)**	**Eligible outcomes**	**Theme**	**Eligible response scale option(s)**	**Raw data extracted (%)**
Kaplan et al. (2005)	Better patient education materials on options	Availability of provisions to discuss risk-reducing options more effectively	N/A – selected outcome from pre-specified list	69.8
	More information on risk reduction options	More education and training	N/A – selected outcome from pre-specified list	69.4
	More evidence to show efficacy of options	Understanding the benefits of primary prevention	N/A – selected outcome from pre-specified list	59.1
	More time with each patient	Availability of provisions to discuss risk-reducing options more effectively	N/A – selected outcome from pre-specified list	49.3
	More formal training in counselling techniques	More education and training	N/A – selected outcome from pre-specified list	34.6
Macdonald et al. (2020)	Clear guidelines/recommendations	Availability of provisions to discuss risk-reducing options more effectively	Strong facilitator	88.0
	If I had better tools to help me identify patients who were suitable	Availability of provisions to discuss risk-reducing options more effectively	Strong facilitator	68.0
	Support from specialists	Peer support	Strong facilitator	64.0
	I expect positive outcomes for women who take risk-reducing medications	Understanding the benefits of primary prevention	Strong facilitator	59.0
	If a patient has a strong family history of breast cancer	Patient has indications of increased breast cancer risk	Strong facilitator	54.0
	Knowing some risk-reducing medications are PBS (Pharmaceutical Benefits Scheme) funded	Knowing some risk-reducing medications are available at a Government-subsidised price	Strong facilitator	54.0
	If it were endorsed as part of my role by the relevant college/peak body	Endorsement as part of role by a professional body	Strong facilitator	53.0
	If the patient has LCIS that increases their risk of breast cancer	Patient has indications of increased breast cancer risk	Strong facilitator	49.0
	If the patient has atypical hyperplasia that increases their risk of breast cancer	Patient has indications of increased breast cancer risk	Strong facilitator	36.0
	Support from my peers	Peer support	Strong facilitator	34.0
	If my medical software prompted me to discuss risk-reducing medications	Availability of provisions to discuss risk-reducing options more effectively	Strong facilitator	33.0
	Sometimes it is easier to discuss risk-reducing medications than bilateral mastectomy	Easier to discuss risk-reducing medications than bilateral mastectomy	Strong facilitator	32.0
	If I knew my colleagues discuss it with their patients	Peer support	Strong facilitator	27.0

	The beneficial effects of risk-reducing medications	Understanding the benefits of primary prevention	Strong facilitator	14.0
Samimi et al. (2020)	The benefits of preventive agents in breast cancer outweigh the risks	Believing that the benefits of preventive agents in breast cancer outweigh the risks	Agree	62.8
Tighe (2009)	Do you believe that the benefits of chemoprevention for breast cancer outweigh the risks?	Believing that the benefits of preventive agents in breast cancer outweigh the risks	Yes	12.4
