# Supplementary material for: Breast Cancer Risk Assessment and Primary Prevention Advice in Primary Care: A Systematic Review of Provider Attitudes and Routine Behaviours
Source: Cancers (Basel). 2021 Aug 18;13(16):4150. doi: 10.3390/cancers13164150 (PMC8394615; doi:10.3390/cancers13164150)
Supplement: Supplementary file 1 [file cancers-13-04150-s001.zip › cancers-1340598-Supplementary material S3.pdf]

Material S3: Summary and detailed results of the quality appraisal using the Mixed Methods Appraisal Tool

Authors (year)	MMAT question				
	1. Is the sampling strategy relevant to address the research question?	2. Is the sample representative of the target population?	3. Are the measurements appropriate?	4. Is the risk of nonresponse bias low?	5. Is the statistical analysis appropriate to answer the research question?
Armstrong et al., (2006)	Somewhat	Yes	Can't tell	Somewhat	Yes
Bankhead et al., (2001)	Yes	Can't tell	Somewhat	Somewhat	Somewhat
Bethea et al., (2008)	Yes	Somewhat	Somewhat	Somewhat	Yes
Bidassie et al., (2020)	Somewhat	Can't tell	Somewhat	Can't tell	Yes
Carroll et al., (2011)	Yes	Somewhat	Somewhat	No	Yes
Casas et al., (2017)	No	No	Can't tell	Somewhat	Yes
Corbelli et al., (2014)	Can't tell	No	Can't tell	Somewhat	Yes
Dekanek et al., (2020)	Somewhat	No	Somewhat	No	Yes
Edwards et al., (2009)	Somewhat	Can't tell	Somewhat	Can't tell	Somewhat
Escher & Sappino (2000)	Somewhat	Can't tell	Can't tell	Yes	Yes
Ganry & Boche (2005)	Yes	Yes	Can't tell	No	Yes
Guerra et al., (2009)	Somewhat	Yes	Can't tell	Somewhat	Yes
Gunn et al., (2018)	Somewhat	No	Can't tell	Somewhat	Yes
Hall et al., (2001)	Somewhat	Can't tell	Somewhat	Somewhat	Yes
Kaplan et al., (2011)	Somewhat	Yes	Somewhat	No	Yes
Khong et al., (2015)	Can't tell	No	Can't tell	No	Yes
Macdonald et al., (2020)	Somewhat	Can't tell	Somewhat	No	Yes
Maimone et al., (2017)	Somewhat	Yes	Can't tell	No	Can't tell
Mainous et al., (2013)	Somewhat	Yes	Can't tell	No	Yes
Nippert et al., (2014)	Somewhat	Yes	Somewhat	No	Yes
Pichert et al., (2003)	Somewhat	Can't tell	Can't tell	No	Can't tell
Sabatino et al., (2007)	No	No	Somewhat	Yes	Yes
Samimi et al., (2020)	Somewhat	Yes	Somewhat	No	Yes
Saunders-Goldson et al., (2018)	No	No	Somewhat	Can't tell	Yes
Summerton & Garrood (1997)	Yes	Somewhat	Somewhat	Somewhat	Can't tell

Tighe et al., (2009)	Somewhat	Somewhat	Somewhat	No	Yes
Walter et al., (2001)	Can’t tell	Can’t tell	Somewhat	Somewhat	Can’t tell
Welkenhuysen & Evers-Kiebooms (2002)	Somewhat	Yes	Somewhat	Yes	Yes
Wilson et al., (2006)	Yes	Somewhat	Somewhat	Somewhat	Yes

**Study:** Armstrong et al., (2006)
**Screening question 1: Are there clear research questions?**
Decision: Yes
**Screening question 2: Do the collected data allow to address the research questions?**
Decision: Yes
**1. Is the sampling strategy relevant to address the research question?**
Decision: Somewhat<br><br>Strengths for this criterion include the source of the sample being relevant to the target population and the use of random stratified sampling. However, the sample frame is biased as only registered members of the American Medical Association could participate.
**2. Is the sample representative of the target population?**
Decision: Yes<br><br>The target population was primary care physicians in the USA. A national survey using random stratified sampling resulted in a sample with representation from all relevant specialties of the target population (internal medicine, obstetrics and gynaecology and family practice). Therefore, the sample is representative of the target population and results generalisable.
**3. Are the measurements appropriate?**
Decision: Can't tell<br><br>The measurements map onto the respective research questions and are therefore appropriate for answering the research question. They are also clearly defined in terms of wording and response scales and there is an acceptable degree of face validity. Items assessing the attitudinal and practice factors were developed based on interviews with primary care physicians and literature about clinical decision making about tamoxifen. However, there is no evidence that the measurements were subject to a comprehensive assessment of reliability and validity nor is there any evidence of the survey being pre-tested prior to data collection. Therefore, a judgement of appropriateness cannot be made with certainty.
**4. Is the risk of nonresponse bias low?**
Decision: Somewhat<br><br>Satisfactory response rate of 47.2%. Respondents did not significantly differ from non-respondents on variables of interest (sex, region of the country, specialty or type of degree). Responders had graduated from medical school more recently than non-responders but this is unlikely to have biased the results.
**5. Is the statistical analysis appropriate to answer the research question?**
Decision: Yes<br><br>Analyses are clearly stated, justified and were appropriate to answer the research question. Alternative categorizations for dichotomization of outcomes were tested and did not change the main result.

**Study:** Bankhead et al., (2001)
**Screening question 1: Are there clear research questions?**
Decision: Yes
**Screening question 2: Do the collected data allow to address the research questions?**
Decision: Yes
**1. Is the sampling strategy relevant to address the research question?**
Decision: Yes<br><br>The source of the sample was relevant to the target population; lists of practice nurses were obtained directly from the Nurse Directors in each health authority where possible and by contacting all GP practices if the data were not available from the health authority. All practice nurses in the four regions were invited to participate. Therefore, the sampling procedure was adequate and unlikely to have introduced bias.
**2. Is the sample representative of the target population?**
Decision: Can't tell<br><br>The target population was UK practice nurses. The sample was drawn from four regions in the UK (three regions of England and one region in Scotland). As no information is reported about the practice landscape in the four regions or socio-demographic characteristics of the sample, a judgement of representativeness cannot be made with certainty.
**3. Are the measurements appropriate?**
Decision: Somewhat<br><br>The measurements map onto the respective research questions and are therefore appropriate for answering the research questions. There is an acceptable degree of face validity. The questionnaire was developed in collaboration with practice nurses and it was piloted. The full response scale for confidence items is not reported. However, there is no evidence that the survey was subject to a comprehensive assessment of reliability and validity.
**4. Is the risk of nonresponse bias low?**
Decision: Somewhat<br><br>Satisfactory response rate of 66%. However, no information is reported about those who did not take part and no analysis of whether respondents were significantly different to non-respondents was conducted.
**5. Is the statistical analysis appropriate to answer the research question?**
Decision: Somewhat<br><br>The analysis appears to be appropriate for answering the research questions but very minimal information is reported beyond the program used (Stata) and the descriptive nature of analysis.

**Study:** Bethea et al., (2008)
**Screening question 1: Are there clear research questions?**
Decision: Yes
**Screening question 2: Do the collected data allow to address the research questions?**
Decision: Yes
**1. Is the sampling strategy relevant to address the research question?**
Decision: Yes<br><br>The source of the sample was relevant to the target population. The four geographical areas were chosen to represent areas that were rural and urban. All practices were sent a letter offering the opportunity to participate in the study. From the 17 practices who expressed an interest in participating, 10 were randomly selected for inclusion. Therefore, the sampling procedure was adequate and unlikely to have introduced bias.
**2. Is the sample representative of the target population?**
Decision: Somewhat<br><br>The target population was UK GPs and practice nurses. Although the four geographical areas were chosen to represent areas that were rural and urban, they were all served by 1 hospital based genetics unit and therefore the results are likely to be limited in generalisability to the target population.
**3. Are the measurements appropriate?**
Decision: Somewhat<br><br>The measurements map onto the respective research questions and are therefore appropriate for answering the research questions. There is an acceptable degree of face validity. The questionnaire had previously been validated in the setting it was being used in and it was developed through a series of primary care-based focus groups. Although the questionnaire had previously been validated in the setting and attempts have been made to improve the content validity, there is no evidence that the survey was subject to a comprehensive assessment of reliability and validity.
**4. Is the risk of nonresponse bias low?**
Decision: Somewhat<br><br>Satisfactory response rate of 59.4%. However, no information is reported about those who did not take part and no analysis of whether respondents were significantly different to non-respondents was conducted.
**5. Is the statistical analysis appropriate to answer the research question?**
Decision: Yes<br><br>Analyses are clearly stated, justified and were appropriate to answer the research question.

**Study:** Bidassie et al., (2020)
**Screening question 1: Are there clear research questions?**
Decision: Yes
**Screening question 2: Do the collected data allow to address the research questions?**
Decision: Yes
**1. Is the sampling strategy relevant to address the research question?**
Decision: Somewhat<br><br>The source of the sample was relevant to the target population but the non-probability sampling procedure is likely to have introduced bias; primary care chiefs at all VA nationwide were contacted and asked to complete the survey and then to forward it to eligible primary care providers at their local VA (snowball sampling).
**2. Is the sample representative of the target population?**
Decision: Can’t tell<br><br>The target population was primary care providers within the Department of Veterans Affairs. The sampling strategy relied on primary care chiefs forwarding the survey on to all eligible primary care providers. The size of the target population is not reported and therefore it is impossible to know how many primary care providers received the invite and in turn make a judgement of how representative the sample is. Nevertheless, generalisability is likely to be limited given the snowball sampling strategy.
**3. Are the measurements appropriate?**
Decision: Somewhat<br><br>The measurements map onto the respective research questions and are therefore appropriate for answering the research questions. The survey has an acceptable degree of face validity. The VA Breast Health Research Group in consultation with survey methodology experts developed the survey. The survey was field tested among primary care providers at one VA to ensure feasibility and validity. However, there is no evidence that the survey was subject to a comprehensive assessment of reliability and validity.
**4. Is the risk of nonresponse bias low?**
Decision: Can’t tell<br><br>The total number of people who received the survey invitation is unknown so a response rate could not be calculated. Therefore risk of non-response bias cannot be determined.
**5. Is the statistical analysis appropriate to answer the research question?**
Decision: Yes<br><br>Analyses are clearly stated, justified and were appropriate to answer the research questions.

**Study:** Carroll et al., (2011)
**Screening question 1: Are there clear research questions?**
Decision: Yes
**Screening question 2: Do the collected data allow to address the research questions?**
Decision: Yes
**1. Is the sampling strategy relevant to address the research question?**
Decision: Yes<br><br>The source of the sample was relevant to the target population; lists of practicing family physicians grouped by practice from the chiefs of Family Medicine at local hospitals. Furthermore, random stratified sampling was used and attempts were made to recruit family physicians from rural and urban areas.
**2. Is the sample representative of the target population?**
Decision: Somewhat<br><br>The target population was Canadian family physicians. The study population consisted of family physicians practising in four locations in Ontario. Although representation from different practice locations was achieved, the majority of physicians practised in large cities (69%) so the sample is likely to have limited generalisability to the target population.
**3. Are the measurements appropriate?**
Decision: Somewhat<br><br>The measurements map onto the respective research questions and are therefore appropriate for answering the research questions. The survey has an acceptable degree of face validity. The clinical scenarios were informed by previous work, the referral guidelines and clinical experience. They were pilot tested with family physicians not involved in the study for comprehension and face validity. The list of competencies for the confidence outcome was derived from the National Coalition for Health Professional Education in Genetics and previous work. However, the authors acknowledge that the outcome measures require psychometric testing of reliability and validity.
**4. Is the risk of nonresponse bias low?**
Decision: No<br><br>Poor response rate of 13%. No examination of whether respondents were significantly different to non-respondents is reported.
**5. Is the statistical analysis appropriate to answer the research question?**
Decision: Yes<br><br>Analyses are clearly stated, justified and were appropriate to answer the research questions.

**Study:** Casas et al., (2017)
**Screening question 1: Are there clear research questions?**
Decision: Yes
**Screening question 2: Do the collected data allow to address the research questions?**
Decision: Yes
**1. Is the sampling strategy relevant to address the research question?**
Decision: No<br><br>The source of the sample was relevant to the target population but the sampling procedure was weak. It is not clear why family medicine and obstetrics and gynaecology clinicians were not included as they are mentioned as a target group for the workshop. Arguably obstetrics and gynaecology clinicians are most likely to counsel women about breast health so their omission is a significant limitation of the sampling strategy.
**2. Is the sample representative of the target population?**
Decision: No<br><br>The target population was practicing clinicians who counsel women about breast health. Participants were recruited from a single hospital affiliated with an academic institution (Boston University Medical Center). Coupled with the omission of relevant clinicians and small sample size, the sample has poor generalisation to the population and selection bias is likely to be present.
**3. Are the measurements appropriate?**
Decision: Can't tell<br><br>The measurements map onto the respective research questions and are therefore appropriate for answering the research questions. The survey has an acceptable degree of face validity. The attitude questions were derived from a review of the literature and in accord with the learning objectives. No evidence of pre-testing the survey prior to data collection is reported. Therefore, a judgement of appropriateness cannot be made with certainty.
**4. Is the risk of nonresponse bias low?**
Decision: Somewhat<br><br>Poor response rate of 9.3%. Twenty-one clinicians who did not attend the educational session participated in the baseline survey (referent group). There was no significant difference between the intervention group and the referent group by gender, years in practice or percentage of female patients in their clinic panels.
**5. Is the statistical analysis appropriate to answer the research question?**
Decision: Yes<br><br>Analyses are clearly stated, justified and were appropriate to answer the research questions.

**Study:** Corbelli et al., (2014)
**Screening question 1: Are there clear research questions?**
Decision: Yes
**Screening question 2: Do the collected data allow to address the research questions?**
Decision: Yes
**1. Is the sampling strategy relevant to address the research question?**
Decision: Can't tell<br><br>Purposive sampling has been used meaning participants have been chosen based on the study's purpose. It is unclear what sample frame was used and how the web-based survey was circulated. Therefore, it is not possible to assess the appropriateness of the sampling strategy with certainty.
**2. Is the sample representative of the target population?**
Decision: No<br><br>The target population was US internists, family physicians and gynaecologist and the resulting sample matched the target population. However, participants were recruited from a single hospital affiliated with an academic institution (University of Pittsburgh Medical Center). The authors acknowledge that their results may have limited generalisability to other regions of the country or to providers who practice in community or rural settings.
**3. Are the measurements appropriate?**
Decision: Can't tell<br><br>The measurements map onto the respective research questions and are therefore appropriate for answering the research questions. There is an acceptable degree of face validity. The survey was adapted from the Breast and Cervical Cancer Screening Questionnaire, part of the National Survey of Primary Care Physicians' Cancer Screening Recommendations and Practices originally conducted by the National Cancer Institute. No evidence of pre-testing the survey prior to data collection is reported. There is no evidence that the adapted survey was subject to a comprehensive assessment of reliability and validity. Therefore, a judgement of appropriateness cannot be made with certainty.
**4. Is the risk of nonresponse bias low?**
Decision: Somewhat<br><br>Satisfactory response rate of 55%. No examination of whether respondents were significantly different to non-respondents is reported.
**5. Is the statistical analysis appropriate to answer the research question?**
Decision: Yes<br><br>Analyses are clearly stated, justified and were appropriate to answer the research questions.

**Study:** Dekanek et al., (2020)
**Screening question 1: Are there clear research questions?**
Decision: Yes
**Screening question 2: Do the collected data allow to address the research questions?**
Decision: Yes
**1. Is the sampling strategy relevant to address the research question?**
Decision: Somewhat<br><br>Purposive sampling has been used meaning participants have been chosen based on the study's purpose. The source of the sample was relevant to the target population; the questionnaire was distributed by email by the Chair of the Department of Obstetrics and Gynaecology and the Director of the Family Medicine Residency Program. This sampling strategy could have introduced bias as participants may have felt compelled to participate and respond in a certain way as the invite was sent by someone in authority.
**2. Is the sample representative of the target population?**
Decision: No<br><br>The target population was US primary care physicians. Participants were recruited from a single hospital affiliated with an academic institution (University of Pittsburgh Medical Center). Coupled with half of the respondents having more than 15 years of experience and the hospital having a subspecialty cancer genetics clinic, the sample has poor generalisation to the population and selection bias is likely to be present.
**3. Are the measurements appropriate?**
Decision: Somewhat<br><br>The measurements map onto the respective research questions and are therefore appropriate for answering the research questions. There is an acceptable degree of face validity. The knowledge questions were based on a previous questionnaire developed in 1999. The opinion questions were designed to reflect the research questions but how these were chosen is not described. The questionnaire was piloted with a focus group of clinicians before data collection. There is no evidence that the measurements were subject to a comprehensive assessment of reliability and validity.
**4. Is the risk of nonresponse bias low?**
Decision: No<br><br>Poor response rate of 25%. No examination of whether respondents were significantly different to non-respondents is reported.
**5. Is the statistical analysis appropriate to answer the research question?**
Decision: Yes<br><br>Analyses are clearly stated, justified and were appropriate to answer the research questions.

**Study:** Edwards et al., (2009)
**Screening question 1: Are there clear research questions?**
Decision: Yes
**Screening question 2: Do the collected data allow to address the research questions?**
Decision: Yes
**1. Is the sampling strategy relevant to address the research question?**
Decision: Somewhat<br><br>The source of the sample is relevant to the target population but the non-probability sampling procedure is likely to have introduced bias; due to cost and travel constraints, the authors distributed surveys via convenience sampling to nurse practitioners attending a national nurse practitioner conference.
**2. Is the sample representative of the target population?**
Decision: Can't tell<br><br>Participants were recruited from one national nurse practitioner conference held in a Midwest US city. The national nature of the conference could have allowed for participation of nurse practitioners across the USA but whether this was the case is not known. Therefore, it is difficult to determine representativeness of the sample but it likely to be limited given the convenience sampling strategy.
**3. Are the measurements appropriate?**
Decision: Somewhat<br><br>The survey was developed in-house by the research team and was reviewed by a group of clinicians including experts in breast cancer risk assessment and prevention and nurse practitioners. The survey was modified and revised based on reviewers' comments and recommendations. The final version was determined to have content validity. The measurements were not subject to a comprehensive assessment of reliability and validity.
**4. Is the risk of nonresponse bias low?**
Decision: Can't tell<br><br>As surveys were handed out and completed in person at the conference, all 175 were returned. It is not possible to calculate a response rate as the authors do not report how many people were approached to complete the survey. Therefore, a judgement of non-response bias cannot be made with certainty.
**5. Is the statistical analysis appropriate to answer the research question?**
Decision: Somewhat<br><br>Minimal information is reported with respect to analysis particularly regarding what statistical calculations were performed. Furthermore, the correlation reported in the results was not specified apriori and is unrelated to a research question.

Study: Escher & Sappino (2000)

Screening question 1: Are there clear research questions?

Decision: Yes

Screening question 2: Do the collected data allow to address the research questions?

Decision: Yes

1. Is the sampling strategy relevant to address the research question?

Decision: Somewhat

Purposive sampling has been used meaning participants have been chosen based on the study's purpose. The source of the sample was relevant to the target population but the sample frame was biased as only registered members of Geneva Medical Association could participate.

2. Is the sample representative of the target population?

Decision: Can't tell

Participants were recruited from one county in Switzerland. The resulting sample appears to match the target population but there is no evidence that a comparison of socio-demographic characteristics between the sample and the target population was performed. Furthermore, it is unclear whether representation from different geographic practice settings was achieved. Therefore, a judgement of representativeness cannot be made with certainty.

3. Are the measurements appropriate?

Decision: Can't tell

The questionnaire was developed in-house by the research team with some items adapted from a questionnaire used in a previous study for a different disease. No attempts at ensuring usability have been described. Furthermore, there is no evidence that the questionnaire was subject to a comprehensive assessment of reliability and validity. Therefore, a judgement of appropriateness cannot be made with certainty.

4. Is the risk of nonresponse bias low?

Decision: Yes

Satisfactory response rate of 65%. Respondents were compared to non-respondents on two variables; specialty and sex. Response rates were similar between the specialities.

5. Is the statistical analysis appropriate to answer the research question?

Decision: Yes

Analyses are clearly stated, justified and were appropriate to answer the research questions.

**Study:** Ganry & Boche (2005)
**Screening question 1: Are there clear research questions?**
Decision: Yes
**Screening question 2: Do the collected data allow to address the research questions?**
Decision: Yes
**1. Is the sampling strategy relevant to address the research question?**
Decision: Yes<br><br>The source of the sample was relevant to the target population; all GPs working in Picardy were invited to participate with the assistance of the Regional Private Doctors Union. Therefore, the sampling procedure was adequate and unlikely to have introduced bias.
**2. Is the sample representative of the target population?**
Decision: Yes<br><br>The target population was GPs in the Picardy region of France. The distribution of the response rate for the three departments within Picardy was comparable and representation was achieved from different geographic practice settings (urban/peri urban and rural zones) indicating that the sample is likely to generalise to the target population.
**3. Are the measurements appropriate?**
Decision: Can't tell<br><br>Very little detail is reported about the development of the questionnaire; it appears to have been developed in-house by the research team and no rationale has been provided for selection of questions. The questionnaire has an acceptable degree of face validity. No attempts at ensuring usability have been described. Furthermore, there is no evidence that the questionnaire was subject to a comprehensive assessment of reliability and validity. Therefore, a judgement of appropriateness cannot be made with certainty.
**4. Is the risk of nonresponse bias low?**
Decision: No<br><br>Poor response rate of 33%. The authors do state that they found no difference in the characteristics of the responders in the discussion but no examination of whether respondents were significantly different to non-respondents is explicitly reported in the results section. Additionally, no reasons for non-responses are reported.
**5. Is the statistical analysis appropriate to answer the research question?**
Decision: Yes<br><br>Analyses are clearly stated, justified and were appropriate to answer the research questions.

**Study:** Guerra et al., (2009)
**Screening question 1: Are there clear research questions?**
Decision: Yes
**Screening question 2: Do the collected data allow to address the research questions?**
Decision: Yes
**1. Is the sampling strategy relevant to address the research question?**
Decision: Somewhat<br><br>Strengths for this criterion include the source of the sample being relevant to the target population and the use of random stratified sampling. However, the sample frame was biased as only registered members of the American Medical Association could participate.
**2. Is the sample representative of the target population?**
Decision: Yes<br><br>The target population was primary care physicians in the USA. The use of a national survey coupled with sample stratification resulted in a sample with representation from all relevant specialties of the target population (internal medicine, obstetrics and gynaecology and family practice). Therefore, the sample was representative of the target population.
**3. Are the measurements appropriate?**
Decision: Can't tell<br><br>The PRECEDE model of health behaviour was used to guide survey development. The survey has an acceptable degree of face validity. Items adapted from other physician surveys were used to assess socio-demographic and practice characteristics. The other three content areas appear to have been developed in-house by the research team and no rationale has been provided for selection of questions. No attempts at ensuring usability have been described. Furthermore, there is no evidence that the questionnaire was not subject to a comprehensive assessment of reliability and validity. Therefore, a judgement of appropriateness cannot be made with certainty.
**4. Is the risk of nonresponse bias low?**
Decision: Somewhat<br><br>Response rate of 48.3%. Respondents did not differ significantly from non-responders on variables of interest (sex, region of the country, specialty or type of degree). Responders had graduated from medical school more recently than non-responders but this is unlikely to have biased the results. Additionally, no reasons for non-responses are reported.
**5. Is the statistical analysis appropriate to answer the research question?**
Decision: Yes<br><br>Analyses are clearly stated, justified and were appropriate to answer the research questions.

**Study:** Gunn et al., (2018)
**Screening question 1: Are there clear research questions?**
Decision: Yes
**Screening question 2: Do the collected data allow to address the research questions?**
Decision: Yes
**1. Is the sampling strategy relevant to address the research question?**
Decision: Somewhat<br><br>The source of the sample was relevant to the target population but the non-probability sampling procedure could have introduced bias; clinical leaders within the divisions of two urban academic, safety net hospitals in Boston sent the survey link to all practising primary care physicians in general internal medicine. Participants may have felt compelled to participate and respond in a certain way as the invite was sent by someone in authority.
**2. Is the sample representative of the target population?**
Decision: No<br><br>The target population was primary care providers practising in Massachusetts. Participants were practicing at two urban academic, safety net hospitals. The authors acknowledge that the academic centre context may have made these providers more likely to be aware of medical evidence. The safety-net status of these practices implies that the providers may have fewer resources and see higher acuity patients with complex social and medical needs. Both of these factors limit the generalisability of the results to the target population.
**3. Are the measurements appropriate?**
Decision: Can't tell<br><br>No information is reported regarding development of the questionnaire. Although the questionnaire has an acceptable degree of face validity, no attempts at ensuring usability have been described. Furthermore, there is no evidence that the questionnaire was subject to a comprehensive assessment of reliability and validity. Therefore, a judgement of appropriateness cannot be made with certainty.
**4. Is the risk of nonresponse bias low?**
Decision: Somewhat<br><br>Satisfactory response rate of 55% but no reasons for non-responses are reported. No examination of whether respondents were significantly different to non-respondents is reported.
**5. Is the statistical analysis appropriate to answer the research question?**
Decision: Yes<br><br>Analyses are clearly stated, justified and were appropriate to answer the research questions.

**Study:** Hall et al., (2001)
**Screening question 1: Are there clear research questions?**
Decision: Yes
**Screening question 2: Do the collected data allow to address the research questions?**
Decision: Yes
**1. Is the sampling strategy relevant to address the research question?**
Decision: Somewhat<br><br>The source of the sample was relevant to the target population and justification for the sampling frame is provided. However, the sample frame was biased as only healthcare professionals belonging to a single management group could participate. The locations of the 16 clinical sites were dispersed geographically providing a population of great demographic variability which is a strength of the sampling strategy.
**2. Is the sample representative of the target population?**
Decision: Can't tell<br><br>The target population was US primary healthcare professionals. Participants all worked in the Twin Cities (Minnesota) area. The resulting sample appears to match the target population but there is no evidence that a comparison of socio-demographic characteristics between the sample and the target population was performed. Furthermore, it is unclear whether the demographic and geographic variability inherent in the sampling frame was achieved. Therefore, a judgement of representativeness cannot be made with certainty.
**3. Are the measurements appropriate?**
Decision: Somewhat<br><br>The survey was developed in-house by the primary investigator. The majority of questions were designed for analysis of the variables identified in each specific aim and for comparison within HCP roles and within areas of practice. The questionnaire has an acceptable degree of face validity. A panel of experts participated in the development process of the survey tool. The survey was then piloted on four health professionals to test for reliability and suggested changes were made to the survey. The author acknowledges that replication of the study is required to demonstrate the reliability and validity of the tool.
**4. Is the risk of nonresponse bias low?**
Decision: Somewhat<br><br>Satisfactory response rate of 61.1% but no reasons for non-responses are reported. No examination of whether respondents were significantly different to non-respondents is reported.
**5. Is the statistical analysis appropriate to answer the research question?**
Decision: Yes<br><br>Analyses are clearly stated, justified and were appropriate to answer the research questions.

**Study:** Kaplan et al., (2011)
**Screening question 1: Are there clear research questions?**
Decision: Yes
**Screening question 2: Do the collected data allow to address the research questions?**
Decision: Yes
**1. Is the sampling strategy relevant to address the research question?**
Decision: Somewhat<br><br>The source of the sample was relevant to the target population and non-probability stratified sampling has been used. Although the sample frame is justified, it is biased as only registered members of the American Medical Association could participate.
**2. Is the sample representative of the target population?**
Decision: Yes<br><br>The target population was primary care physicians in California. A large state-wide sample stratified by primary care speciality was collected and representation from different practice settings was achieved. Therefore, the sample is representative of the target population and results generalisable.
**3. Are the measurements appropriate?**
Decision: Somewhat<br><br>The survey was a questionnaire adapted from the physician survey literature, pretested among practising physicians and revised accordingly. The survey has an acceptable degree of face validity. There is no evidence that the questionnaire was subject to a comprehensive assessment of reliability and validity.
**4. Is the risk of nonresponse bias low?**
Decision: No<br><br>Satisfactory response rate of 50%. An examination of whether respondents differed significantly from non-respondents revealed that significantly more women and obstetrician/gynaecologists responded. The authors acknowledge that their findings regarding risk-reduction practices may be overestimated as a result.
**5. Is the statistical analysis appropriate to answer the research question?**
Decision: Yes<br><br>Analyses are clearly stated, justified and were appropriate to answer the research questions.

**Study:** Khong et al., (2015)
**Screening question 1: Are there clear research questions?**
Decision: Yes
**Screening question 2: Do the collected data allow to address the research questions?**
Decision: Yes
**1. Is the sampling strategy relevant to address the research question?**
Decision: Can't tell<br><br>Purposive sampling has been used meaning participants have been chosen based on the study's purpose. The source of the sample was relevant to the target population but the exact sampling frame used is unclear. Therefore, it is not possible to assess the appropriateness of the sampling strategy with certainty.
**2. Is the sample representative of the target population?**
Decision: No<br><br>The target population was primary care physicians in California. Participants were recruited from a single institution affiliated with an academic medical centre and therefore results are unlikely to generalise to the population.
**3. Are the measurements appropriate?**
Decision: Can't tell<br><br>Very minimal information is provided about the survey beyond question format and response options. No rationale is provided for selection of questions. Although the questionnaire has an acceptable degree of face validity, no attempts at ensuring usability have been described. Furthermore, there is no evidence that the questionnaire was subject to a comprehensive assessment of reliability and validity. Therefore, a judgement of appropriateness cannot be made with certainty.
**4. Is the risk of nonresponse bias low?**
Decision: No<br><br>Response rate of 45% and no examination of whether respondents were significantly different to non-respondents is reported.
**5. Is the statistical analysis appropriate to answer the research question?**
Decision: Yes<br><br>Analyses are clearly stated, justified and were appropriate to answer the research questions.

**Study:** Macdonald et al., (2020)
**Screening question 1: Are there clear research questions?**
Decision: Yes
**Screening question 2: Do the collected data allow to address the research questions?**
Decision: Yes
**1. Is the sampling strategy relevant to address the research question?**
Decision: Somewhat<br><br>The source of the sample was relevant to the target population; eligible clinicians were family physicians or breast surgeons involved in the care of women recruited to the Kathleen Cuningham Foundation Consortium for Research into Familial Breast Cancer cohort (kConFab). The resulting sample is therefore biased towards healthcare professionals who are likely to be more familiar with the management of women at high risk of developing breast cancer which may have affected the results.
**2. Is the sample representative of the target population?**
Decision: Can't tell<br><br>The target population was Australian family physicians and breast surgeons. Although the sample recruited appears to match the target population, no information about socio-demographic characteristics or practice setting is reported. Therefore, a judgement of representativeness cannot be made with certainty.
**3. Are the measurements appropriate?**
Decision: Somewhat<br><br>A literature review and focus group interviews were undertaken with family physicians and breast surgeons to inform survey development. The survey has an acceptable degree of face validity and was developed using the Theoretical Domains Framework. However, there is no evidence that the survey was subject to a comprehensive assessment of reliability and validity.
**4. Is the risk of nonresponse bias low?**
Decision: No<br><br>Response rate of 42% and no examination of whether respondents were significantly different to non-respondents is reported.
**5. Is the statistical analysis appropriate to answer the research question?**
Decision: Yes<br><br>Analyses were descriptive in nature (numbers and percentages) which was an appropriate approach given the research questions.

**Study:** Maimone et al., (2017)
**Screening question 1: Are there clear research questions?**
Decision: Yes
**Screening question 2: Do the collected data allow to address the research questions?**
Decision: Yes
**1. Is the sampling strategy relevant to address the research question?**
Decision: Somewhat<br><br>Purposive sampling has been used meaning participants have been chosen based on the study's purpose. The source of the sample was relevant to the target population and the survey was distributed via email but the exact sampling frame used is unclear. Attempts were made to recruit primary care professionals from rural and urban areas which is a strength of the sampling strategy.
**2. Is the sample representative of the target population?**
Decision: Yes<br><br>The target population was Mayo Clinic primary care physicians. Participation from Mayo Clinic sites involved referring providers from three primary campuses in Minnesota, Florida and Arizona, as well as smaller satellite locations. Representation was achieved from different geographic practice settings. Therefore, the sample is representative of the target population and results generalisable.
**3. Are the measurements appropriate?**
Decision: Can't tell<br><br>Very minimal information is provided about the development of the survey beyond it being created by breast imaging radiologists. No rationale is provided for selection of questions. Although the questionnaire has an acceptable degree of face validity, no attempts at ensuring usability have been described. Furthermore, there is no evidence that the questionnaire was subject to a comprehensive assessment of reliability and validity. Therefore, a judgement of appropriateness cannot be made with certainty.
**4. Is the risk of nonresponse bias low?**
Decision: No<br><br>Response rate of 44%. No examination of whether respondents differed significantly from non-respondents is reported.
**5. Is the statistical analysis appropriate to answer the research question?**
Decision: Can't tell<br><br>Descriptive statistics only which appear appropriate for answering the research questions. However, a judgement of appropriateness cannot be made with certainty as there is no analysis section included in the paper.

**Study:** Mainous et al., (2013)
**Screening question 1: Are there clear research questions?**
Decision: Yes
**Screening question 2: Do the collected data allow to address the research questions?**
Decision: Yes
**1. Is the sampling strategy relevant to address the research question?**
Decision: Somewhat<br><br>Purposive sampling has been used meaning participants have been chosen based on the study's purpose. The source of the sample was relevant to the target population. However, the sample frame was biased as only registered members of the four major US academic family medicine organisations could participate.
**2. Is the sample representative of the target population?**
Decision: Yes<br><br>The target population was academic family physicians. A large sample was drawn from the four major US academic family medicine organisations and the resulting sample matched the target population. Therefore, the sample is representative of the target population and results generalisable.
**3. Are the measurements appropriate?**
Decision: Can't tell<br><br>The survey questions were developed following a review of the literature to identify key concepts and issues suggesting the need for additional knowledge. Although the questionnaire has an acceptable degree of face validity, no attempts at ensuring usability have been described. Furthermore, there is no evidence that the questionnaire was subject to a comprehensive assessment of reliability and validity. Therefore, a judgement of appropriateness cannot be made with certainty.
**4. Is the risk of nonresponse bias low?**
Decision: No<br><br>Response rate of 45.1%. The authors state that it was not possible to conduct meaningful analyses regarding differences between respondents and non-respondents due to the large amount of missing data on demographic variables in the membership database.
**5. Is the statistical analysis appropriate to answer the research question?**
Decision: Yes<br><br>Analyses are clearly stated, justified and were appropriate to answer the research questions.

**Study:** Nippert et al., (2014)
**Screening question 1: Are there clear research questions?**
Decision: Yes
**Screening question 2: Do the collected data allow to address the research questions?**
Decision: Yes
**1. Is the sampling strategy relevant to address the research question?**
Decision: Somewhat<br><br>The sampling methods are reported in their sister paper (Den Heijer et al., 2013). Strengths for this criterion include the sources of the samples being relevant to the target population and random samples have been drawn. For some participant groups, the best available lists were used as the sample frame. However, the authors do not report how the sample of UK GPs was obtained and the Dutch GPs were drawn from the membership list of the Dutch Society of GPs, a paid association resulting in a biased sample.
**2. Is the sample representative of the target population?**
Decision: Yes<br><br>The target population was GPs and breast surgeons in four countries. Across all four countries, representation from different geographic practice settings was achieved (inner city, middle sized or small town and town in rural area). Furthermore, participants were recruited using national surveys and the resulting sample matched the target population. Therefore, the sample is representative of the target population and results generalisable.
**3. Are the measurements appropriate?**
Decision: Somewhat<br><br>Information about the development of the questionnaire is reported in their sister paper (Den Heijer et al., 2013). The measurements map onto the research question and are therefore appropriate for answering it. There is an acceptable degree of face validity. The questionnaire was developed following discussions with members of the working group. It was piloted in 10 GPs and 10 specialists in each participating country and feedback was used to refine the questionnaire further. There is no evidence that the questionnaire was subject to a comprehensive assessment of reliability and validity.
**4. Is the risk of nonresponse bias low?**
Decision: No<br><br>Poor response rate of 33%. No examination of whether respondents were significantly different to non-respondents is reported.
**5. Is the statistical analysis appropriate to answer the research question?**
Decision: Yes<br><br>Analyses are clearly stated, justified and were appropriate to answer the research questions.

**Study:** Pichert et al., (2003)
**Screening question 1: Are there clear research questions?**
Decision: Yes
**Screening question 2: Do the collected data allow to address the research questions?**
Decision: Yes
**1. Is the sampling strategy relevant to address the research question?**
Decision: Somewhat<br><br>The source of the sample was relevant to the target population. All relevant primary care specialties are included in the sample frame but no justification is provided for limiting the sample to physicians working in private practice. The sample frame was a pharmaceutical company mailing list obtained from a market research institute meaning that only physicians signed up to this mailing list could participate biasing the sample.
**2. Is the sample representative of the target population?**
Decision: Can't tell<br><br>The target population was primary care physicians in the German speaking part of Switzerland. The resulting sample appears to match the target population but there is no evidence that a comparison of socio-demographic characteristics between the sample and the target population was performed. It is also unclear whether representation from different practice locations was achieved. Therefore, a judgement of representativeness cannot be made with certainty.
**3. Are the measurements appropriate?**
Decision: Can't tell<br><br>No information is reported regarding the development of the measures. The measures appear appropriate for answering the research questions and have an acceptable degree of face validity. However, no evidence of ensuring usability have been described. Furthermore, there is no evidence that the questionnaire was subject to a comprehensive assessment of reliability and validity. Therefore, a judgement of appropriateness cannot be made with certainty.
**4. Is the risk of nonresponse bias low?**
Decision: No<br><br>Response rate of 45%. No examination of whether respondents were significantly different to non-respondents is reported.
**5. Is the statistical analysis appropriate to answer the research question?**
Decision: Can't tell<br><br>Descriptive statistics only which appear appropriate for answering the research questions. However, a judgement of appropriateness cannot be made with certainty as there is no analysis section included in the paper.

**Study:** Sabatino et al., (2007)
**Screening question 1: Are there clear research questions?**
Decision: Yes
**Screening question 2: Do the collected data allow to address the research questions?**
Decision: Yes
**1. Is the sampling strategy relevant to address the research question?**
Decision: No<br><br>Purposive sampling has been used meaning participants have been chosen based on the study's purpose. The sample frame was providers in the general medicine outpatient practice or 1 of 15 affiliated community practices. Relevant clinicians such as family practitioners and gynaecologists have been omitted from the sample frame and no justification is provided for why this decision was made. In the absence of justification, omission of primary care specialties most likely to counsel women about breast health is a significant limitation of the sampling strategy.
**2. Is the sample representative of the target population?**
Decision: No<br><br>The target population was US primary care providers. Participants were recruited from a single urban hospital affiliated with an academic institution (Beth Israel Deaconess Medical Center in Boston). The authors acknowledge that generalisability to other specialties, regions and clinical or non-urban settings may be limited. Coupled with the omission of relevant clinicians the sample has poor generalisation to the population and selection bias is likely to be present.
**3. Are the measurements appropriate?**
Decision: Somewhat<br><br>The measurements map onto the respective research questions and are therefore appropriate for answering the research question. They are also clearly defined in terms of wording and response scales and there is an acceptable degree of face validity. The questionnaire was reviewed by two groups of physicians and pre-tested among seven practicing physicians. However, there is no evidence that the survey was subject to a comprehensive assessment of reliability and validity.
**4. Is the risk of nonresponse bias low?**
Decision: Yes<br><br>Satisfactory response rate of 53%. Respondents did not differ significantly from non-responders on variables of interest (sex, training level or practice setting).
**5. Is the statistical analysis appropriate to answer the research question?**
Decision: Yes<br><br>Analyses are clearly stated, justified and were appropriate to answer the research questions. They assessed the potential confounding of nurse practitioners.

**Study:** Samimi et al., (2020)
**Screening question 1: Are there clear research questions?**
Decision: Yes
**Screening question 2: Do the collected data allow to address the research questions?**
Decision: Yes
**1. Is the sampling strategy relevant to address the research question?**
Decision: Somewhat<br><br>Purposive sampling has been used meaning participants have been chosen based on the study's purpose. The source of the sample was relevant to the target population but the sample frame was biased as participants were recruited from an existing opt-in health care provider panel developed and maintained by a health care market research firm. To become a panel member, healthcare providers also needed to be a member of the American Medical Association.
**2. Is the sample representative of the target population?**
Decision: Yes<br><br>The target population was US primary care physicians. All relevant primary care specialties were recruited. Information about geographic practice setting is reported in Supplementary File 2 and this indicates that representation was achieved from all geographic practice settings (urban, suburban, rural and geographically isolated/remote). The sample is therefore representative of the target populations and results generalisable.
**3. Are the measurements appropriate?**
Decision: Somewhat<br><br>Information about survey development is reported in their sister paper (Samimi et al., 2019).The survey was developed based on previous studies by the authors and a literature review. The survey was initially tested using semi structured Web-assisted cognitive interviews with 9 US-based primary care physicians to evaluate whether they understood the survey questions and could respond as intended. The final questionnaire was developed based on this feedback. The survey has an acceptable degree of face validity. However, there is no evidence that the questionnaire was subject to a comprehensive assessment of reliability and validity.
**4. Is the risk of nonresponse bias low?**
Decision: No<br><br>Poor response rate of 12% and no examination of whether respondents were significantly different to non-respondents is reported.
**5. Is the statistical analysis appropriate to answer the research question?**
Decision: Yes<br><br>Analyses are clearly stated, justified and were appropriate to answer the research questions.

**Study:** Saunders-Goldson et al., (2018)
**Screening question 1: Are there clear research questions?**
Decision: Yes
**Screening question 2: Do the collected data allow to address the research questions?**
Decision: Yes
**1. Is the sampling strategy relevant to address the research question?**
Decision: No<br><br>The source of the sample was relevant to the target population but the sampling procedure was weak. Very little information is reported about the sampling strategy beyond convenience sampling being used. The accessible population of first through fourth year resident physicians in internal medicine and family practice differs from the target population as obstetrics and gynaecology residents have been omitted and no justification is provided for why this decision was made. In the absence of justification, omission of the primary care speciality most likely to counsel women about breast health is a significant limitation of the sampling strategy.
**2. Is the sample representative of the target population?**
Decision: No<br><br>The target population was health care providers working in primary care settings. Participants were recruited from a medical school associated with an Ambulatory Indigent Care Center located in an urban city in the Northeastern United States. Coupled with the omission of relevant clinicians and small sample size, the sample has poor generalisation to the population and selection bias is likely to be present.
**3. Are the measurements appropriate?**
Decision: Somewhat<br><br>Knowledge was measured using a survey instrument (the Breast Cancer Risk Assessment Knowledge Tool) developed by experts in the field of hereditary breast cancer and genetics. The tool has an established content validity and reliability with a Cronbach's alpha of 0.89. The attitudes measure was developed by a team of genetic counsellors specialising in hereditary cancers and primary care physicians and piloted by clinicians in the areas of genetic counselling, oncology, surgery, and family practice (information reported in sister paper – Koil et al., 2003). Both measures have an acceptable degree of face validity. However, there is no evidence that the attitudes measure was subject to a comprehensive assessment of reliability and validity.
**4. Is the risk of nonresponse bias low?**
Decision: Can't tell<br><br>It is reported that fifty surveys were distributed to physician residents of which 22 met the inclusionary criteria. It is not possible to compare respondents and non-respondents due to the convenience sampling procedure. Therefore, a judgement of non-response bias cannot be made with certainty.
**5. Is the statistical analysis appropriate to answer the research question?**
Decision: Yes<br><br>Analyses are clearly stated, justified and were appropriate to answer the research questions.

**Study:** Summerton & Garrood (1997)
**Screening question 1: Are there clear research questions?**
Decision: Yes
**Screening question 2: Do the collected data allow to address the research questions?**
Decision: Yes
**1. Is the sampling strategy relevant to address the research question?**
Decision: Yes<br><br>The source of the sample was relevant to the target population; complete list of GPs practising within the Calderdale and Kirklees Health Authority area. Therefore, the sampling procedure was adequate and unlikely to have introduced bias.
**2. Is the sample representative of the target population?**
Decision: Somewhat<br><br>The target population was UK GPs. The sample was recruited from one health authority in the UK (Calderdale and Kirklees Health Authority). The authors report that 37% of participants worked in urban practices. Whether there was sufficient representation across different geographic practice settings is not reported so generalisability to the UK may be limited. However, sample representativeness was assessed by comparing the GPs who responded to the survey with known demographic variables among British GPs. The results indicated the generally representative nature of the study
**3. Are the measurements appropriate?**
Decision: Somewhat<br><br>The measurements map onto the respective research questions and are therefore appropriate for answering the research questions. Minimal information is reported about the development of the questionnaire. It was piloted among a group of GPs with the results suggesting that the questionnaire exhibited face validity and expert opinions were also supportive of the content. However, there is no evidence that the questionnaire was subject to a comprehensive assessment of reliability and validity.
**4. Is the risk of nonresponse bias low?**
Decision: Somewhat<br><br>Satisfactory response rate of 66.3%. No examination of whether respondents were significantly different to non-respondents is reported.
**5. Is the statistical analysis appropriate to answer the research question?**
Decision: Can't tell<br><br>Descriptive statistics only which appear appropriate for answering the research questions. However, a judgement of appropriateness cannot be made with certainty as there is no analysis section included in the paper.

**Study:** Tighe et al., (2009)
**Screening question 1: Are there clear research questions?**
Decision: Yes
**Screening question 2: Do the collected data allow to address the research questions?**
Decision: Yes
**1. Is the sampling strategy relevant to address the research question?**
Decision: Somewhat<br><br>Strengths for this criterion include the source of the sample being relevant to the target population and the use of random stratified sampling. The sample frame was drawn from the Canadian Medical Directory which is the largest and best available list capturing the majority of doctors practising family medicine in Canada. However, the authors state that coverage may be as low as 69%. Another strength of the sampling strategy was the attempt to achieve proportionate representation from each province by taking into account the percent of the total Canadian population in that province.
**2. Is the sample representative of the target population?**
Decision: Somewhat<br><br>The target population was Canadian family physicians. The resulting sample matched the target population and proportionate representation across all geographic areas in Canada was achieved. However, statistically significant differences were seen in response by gender, province/territory and medical experience which the authors acknowledge may affect the generalisability of the results.
**3. Are the measurements appropriate?**
Decision: Somewhat<br><br>The measurements map onto the respective research questions and are therefore appropriate for answering the research questions. Minimal information is reported about the development of the questionnaire. A focus group session was held with four family physicians in order to pilot the survey and revisions were made. The questionnaire has an acceptable degree of face validity. However, there is no evidence that the questionnaire was subject to a comprehensive assessment of reliability and validity.
**4. Is the risk of nonresponse bias low?**
Decision: No<br><br>Poor response rate of 38.2%. Responders and non-responders did not differ by language or graduation country. However, they did vary significantly by province and number of years practicing. Responders were also more likely to be female. The authors acknowledge that female physicians tend to be more active about prevention so it is likely that rates of prevention knowledge and practices are overestimated.
**5. Is the statistical analysis appropriate to answer the research question?**
Decision: Yes<br><br>Analyses are clearly stated, justified and were appropriate to answer the research questions.

**Study:** Walter et al., (2001)
**Screening question 1: Are there clear research questions?**
Decision: Yes
**Screening question 2: Do the collected data allow to address the research questions?**
Decision: Yes
**1. Is the sampling strategy relevant to address the research question?**
Decision: Can't tell<br><br>The source of the sample was relevant to the target population; all GPs and practice nurses in the 66 practices of the Cambridge and Huntingdon Health Authority were invited to participate. However, the authors do not explicitly state the sample frame used to ensure that all eligible GPs and practice nurses were invited. Therefore, it is not possible to assess the appropriateness of the sampling strategy with certainty.
**2. Is the sample representative of the target population?**
Decision: Can't tell<br><br>The target population was UK GPs and practice nurses. The sample was recruited from one health authority in the UK. Although the sample had similar demographic characteristics to the Cambridge and Huntingdon Health Authority, it differed from the national picture where there are more male doctors. The composition of the county is not reported so it is unclear whether representation from different geographic practice settings UK was achieved. Therefore, a judgement of representativeness cannot be made with certainty.
**3. Are the measurements appropriate?**
Decision: Somewhat<br><br>Questionnaire development was informed by a review of published work. It was piloted among 11 practice teams in South Bedfordshire with the indicated modifications made. The questionnaire has an acceptable degree of face validity. However, there is no evidence that the questionnaire was subject to a comprehensive assessment of reliability and validity.
**4. Is the risk of nonresponse bias low?**
Decision: Somewhat<br><br>Satisfactory response rate of 69%. No examination of whether respondents were significantly different to non-respondents is reported.
**5. Is the statistical analysis appropriate to answer the research question?**
Decision: Can't tell<br><br>The analysis appears to be appropriate for answering the research questions but very minimal information is reported in the form of a generic, vague sentence stating that parametric and non-parametric statistics were used as appropriate. Therefore, a judgement of appropriateness cannot be made with certainty.

**Study:** Welkenhuysen & Evers-Kiebooms (2002)
**Screening question 1: Are there clear research questions?**
Decision: Yes
**Screening question 2: Do the collected data allow to address the research questions?**
Decision: Yes
**1. Is the sampling strategy relevant to address the research question?**
Decision: Somewhat<br><br>Strengths for this criterion include the source of the sample being relevant to the target population and the use of random sampling. However, the sample frame was biased as it only contained registered members of the Scientific Association of Flemish General Practitioners, an association which only half of the GPs in Flanders are members of.
**2. Is the sample representative of the target population?**
Decision: Yes<br><br>The target population was GPs in Flanders. A comparison of respondents with the target population of Flemish GPs was conducted which demonstrated that there was no differences in gender or year of graduation so the resulting sample matched the target population. Furthermore, representation from different geographic practice locations was achieved (town and village). Therefore, the sample is representative of the target population and results generalisable.
**3. Are the measurements appropriate?**
Decision: Somewhat<br><br>Members of the Academic Centre for General Practice Medicine of the University of Leuven were involved in the development of the questionnaires. Questionnaires were thoroughly pilot tested in a group of 33 GPs and have an acceptable degree of face validity. However, there is no evidence the questionnaires were subject to a comprehensive assessment of reliability and validity.
**4. Is the risk of nonresponse bias low?**
Decision: Yes<br><br>Response rate of 51.8%. The group of responders did not differ from the group of non-responders with regard to gender or year of graduation.
**5. Is the statistical analysis appropriate to answer the research question?**
Decision: Yes<br><br>Analyses are clearly stated, justified and were appropriate to answer the research questions.

**Study:** Wilson et al., (2006)
**Screening question 1: Are there clear research questions?**
Decision: Yes
**Screening question 2: Do the collected data allow to address the research questions?**
Decision: Yes
**1. Is the sampling strategy relevant to address the research question?**
Decision: Yes<br><br>The source of the sample was relevant to the target population. All practices within the study setting were eligible for inclusion. Furthermore, there were attempts to achieve representation from different geographic practice settings which is a strength of the sampling procedure.
**2. Is the sample representative of the target population?**
Decision: Somewhat<br><br>The target population was UK GPs. The accessible population was GPs working in the Grampian region of Scotland. The attempts to achieve representation from different geographic practice settings (urban and rural) were successful which improves the generalisability of the sample. Overall, the resulting sample matched the target population but the representativeness could have been improved if the sampling strategy wasn't limited to one specific region or the authors could have conducted a comparison of socio-demographic characteristic between the sample and the target population to assess generalisability beyond the region.
**3. Are the measurements appropriate?**
Decision: Somewhat<br><br>No information is reported regarding the development of the measures. The measures appear appropriate for answering the research questions and have an acceptable degree of face validity. The questionnaire was developed and piloted in two practices. However, there is no evidence that the questionnaire was subject to a comprehensive assessment of reliability and validity.
**4. Is the risk of nonresponse bias low?**
Decision: Somewhat<br><br>Satisfactory response rate to baseline survey of 78.6%. No examination of whether respondents were significantly different to non-respondents is reported.
**5. Is the statistical analysis appropriate to answer the research question?**
Decision: Yes<br><br>Analyses are clearly stated, justified and were appropriate to answer the research questions.
