# Supplementary material for: Breast Cancer Risk Assessment and Primary Prevention Advice in Primary Care: A Systematic Review of Provider Attitudes and Routine Behaviours
Source: Cancers (Basel). 2021 Aug 18;13(16):4150. doi: 10.3390/cancers13164150 (PMC8394615; doi:10.3390/cancers13164150)
Supplement: Supplementary file 1 [file cancers-13-04150-s001.zip › cancers-1340598-Supplementary table S1.pdf]

Table S1: A list of 95 excluded studies and reasons for exclusion

**Reference**	**Reason for exclusion**
Abda, N., et al. (2017). Knowledge, Attitudes, and Preventive Practice Towards Breast Cancer among General Practitioner Health Professionals in Morocco. Asian Pacific Journal of Cancer Prevention **18**(4): 963-968.	No eligible outcome measures<br>*Knowledge only*
Bellcross, C. A., et al. (2011). Awareness and utilization of BRCA1/2 testing among U.S. primary care physicians. American Journal of Preventive Medicine **40**(1): 61-66.	No eligible outcome measures<br>*Behaviour outcomes not eligible as not routine behaviour (specifies a timeframe (in the past year))*
Chamberlain, R. M., et al. (1995). Improving residents' knowledge of cancer prevention: Are physicians prepared for prevention?. Journal of Cancer Education **10**(1): 9-13.	No eligible outcome measures<br>*Knowledge only*
Cockburn, J., et al. (1989). Encouraging attendance at screening mammography: knowledge, attitudes and intentions of general practitioners. The Medical Journal of Australia **151**(7): 391-396.	No eligible outcome measures<br>*Knowledge only*
Cohn, J., et al. (2015). Physician risk assessment knowledge regarding BRCA genetics testing. Journal of Cancer Education **30**(3): 573-579.	No eligible outcome measures<br>*Knowledge only*
Edwards, Q. T., & Seibert, D. (2010). Pre-and post-test evaluation of a breast cancer risk assessment program for nurse practitioners. Journal of the American Academy of Nurse Practitioners **22**(7): 376-381.	No eligible outcome measures<br>*Knowledge only*
Haas, J. S., et al. (2004). Do physicians tailor their recommendations for breast cancer risk reduction based on patient's risk?. Journal of General Internal Medicine **19**(4): 302-309.	No eligible outcome measures<br>*Behaviour outcome ineligible as clinical scenario*

Hapgood, R., et al. (2002). Breast cancer genetics in primary care which GPs most accurately categorise patients at low risk?. The European Journal of General Practice **8**(4): 146-150.	No eligible outcome measures<br>*Behaviour outcome ineligible as clinical scenario*
Johnson, K. M., et al. (1998). Inner city primary care providers' breast cancer screening knowledge: implications for intervention. Journal of Community Health **23**(1): 1-14.	No eligible outcome measures<br>*Knowledge only*
Julian-Reynier, C., et al. (2015). General Practitioners and Breast Surgeons in France, Germany, Netherlands and the UK show variable breast cancer risk communication profiles. BMC Cancer **15**(1): 1-9.	No eligible outcome measures<br>*Ineligible as risk communication practices (what was discussed about risk and how this was presented i.e. absolute, relative risks etc.)*
Kirby, S., & Hegarty, J. (2010). Breast awareness within an intellectual disability setting. European Journal of Oncology Nursing **14**(4): 328-336.	No eligible outcome measures<br>*Knowledge only*
Klitzman, R., et al. (2013). Attitudes and practices among internists concerning genetic testing. *Journal of Genetic Counseling* **22**(1), 90-100.	No eligible outcome measures<br>*Behaviour outcomes not eligible as not routine behaviour (specifies a timeframe (in the last 6 months))*
Koil, C. E., et al. (2003). Differences in physician referral practices and attitudes regarding hereditary breast cancer by clinical practice location. Genetics in Medicine **5**(5): 364-369.	No eligible outcome measures<br>*Behaviour outcomes not eligible as not routine behaviour (% who have ever referred)*
Nair, N., et al. (2017). Georgia primary care providers' knowledge of hereditary breast and ovarian cancer syndrome. Journal of Cancer Education **32**(1): 119-124.	No eligible outcome measures<br>*Knowledge only*
Obeidat, N. A., et al. (2017). Are Jordanian primary healthcare practitioners fulfilling their potential in cancer prevention and community health? Findings from a cross-sectional survey. BMJ Open **7**(4): e015269.	No eligible outcome measures<br>*Knowledge only*

Pirdehghan, A., et al. (2019). Assessing breast cancer knowledge among Iranian physicians. International Journal of Cancer Management **12**(4): e85822.	No eligible outcome measures<br>*Knowledge only*
Rose, P. W., et al. (2001). Referral of patients with a family history of breast/ovarian cancer—GPs' knowledge and expectations. Family Practice **18**(5): 487-490.	*No eligible outcome measures*<br>*Behaviour outcome ineligible as clinical scenario*
Samimi, G., et al. (2020). Cancer Prevention in Primary Care: Perception of Importance, Recognition of Risk Factors and Prescribing Behaviors. The American Journal of Medicine **133**(6): 723-732.	No eligible outcome measures<br>*Behaviour outcomes not eligible as not routine behaviour (specifies a timeframe (in the last 12 months))*
Smith, S. G., et al. (2017). Prescribing tamoxifen in primary care for the prevention of breast cancer: a national online survey of GPs' attitudes. British Journal of General Practice **67**(659): e414-e427.	No eligible outcome measures<br>*Behaviour outcome ineligible as hypothetical case study*
Soyer, M. T., et al. (2007). Breast cancer awareness and practice of breast self-examination among primary health care nurses: influencing factors and effects of an in-service education. Journal of Clinical Nursing **16**(4): 707-715.	No eligible outcome measures<br>*Knowledge only*
Venkatesh, G. M., & Sundar, M. (2020). Breast Cancer Screening: Are 'At Risk Population' Known by Public Health Nurse Practitioners?. Indian Journal of Public Health Research & Development **11**(1): 369-373.	No eligible outcome measures<br>*Knowledge only*
Watson, E., et al. (2001). A study of GP referrals to a family cancer clinic for breast/ovarian cancer. Family Practice **18**(2): 131-134.	No eligible outcome measures<br>*Behaviour outcome ineligible as reflection on previous clinical case*
Weston, C., et al. (2018). The impact of interprofessional education on family nurse practitioner students' and family medicine residents' knowledge and confidence in screening for breast and cervical cancer. Journal of the American Association of Nurse Practitioners **30**(9): 511-518.	No eligible outcome measures<br>*Confidence outcome ineligible as about secondary prevention (breast screening)*

Yong, M. C., et al. (2003). The importance of paternal family history in hereditary breast cancer is underappreciated by health care professionals. Oncology **64**(3): 220-226.	No eligible outcome measures<br>*Knowledge only*
Yousuf, S. A., et al. (2012). Do Saudi nurses in primary health care centres have breast cancer knowledge to promote breast cancer awareness?. Asian Pacific Journal of Cancer Prevention **13**(9): 4459-4464.	No eligible outcome measures<br>*Knowledge only*
Abittan, B., et al. (2018). Provider-Patient Communication of Personal Breast Cancer Risk (BCR): Providers' Beliefs. Obstetrics & Gynecology **131** 145S. 66th Annual Clinical and Scientific Meeting of the American College of Obstetricians and Gynecologists, USA.	Conference proceeding
Blouin-Bougie J., et al. (2014). Breast cancer risk prediction and risk communication practices: Evidence from a Canadian survey. Current Oncology **21**(2): e385. 5th International Symposium on Hereditary Breast and Ovarian Cancer, Montreal, Canada.	Conference proceeding
Bryan T., et al. (2013). Education in delivering patient-centered care: Provider comfort level in counseling women ages 40-49 regarding breast cancer screening options. Journal of Investigative Medicine **61**(2): 524. American Federation for Medical Research Southern Regional Meeting, New Orleans, USA.	Conference proceeding
Collins I.M., et al. (2013). Development of a tailored, computerized, breast cancer risk assessment and decision support tool: What do clinicians want?. Journal of Clinical Oncology **31**(15 SUPPL. 1). 2013 Annual Meeting of the American Society of Clinical Oncology, Chicago, USA.	Conference proceeding
Corbelli J., & McNeil, M. (2013). Missed opportunities for breast cancer prevention among primary care providers. Journal of General Internal Medicine **28**(SUPPL. 1): S122. 36th Annual Meeting of the	Conference proceeding

Society of General Internal Medicine, Denver, USA.	
Dyer H.G., et al. (2014). Improving early detection and diagnosis of breast cancer in the Commonwealth of Dominica. Asia-Pacific Journal of Clinical Oncology **10**(SUPPL. 9): 122. 2014 World Cancer Congress. Melbourne, Australia.	Conference proceeding
Edwards Q.T., & Seibert, D. (2009). B.C.R.P - Bringing choices in reach for patients: A breast cancer risk assessment program for nurse practitioners. American Journal of Clinical Oncology **32**(5): 552. 19th Annual National Interdisciplinary Breast Center Conference of the National Consortium of Breast Centers, Las Vegas, USA.	Conference proceeding
Gunn C.M., et al. (2016) Primary care provider experience with breast density legislation in Massachusetts. Journal of General Internal Medicine **31**(2 SUPPL. 1): S363-S364). 39th Annual Meeting of the Society of General Internal Medicine, Florida, USA.	Conference proceeding
Larson S.L., et al. (2016). Reducing breast cancer risk: Why aren't providers screening and prescribing?. Pharmacoepidemiology and Drug Safety **25(**SUPPL. 3): 343-344. 32nd International Conference on Pharmacoepidemiology and Therapeutic Risk Management, Ireland.	Conference proceeding
Lauro C.F., et al. (2014). Breast cancer risk assessment and chemoprevention: Results of a survey. International Journal of Radiation Oncology Biology Physics **90**(1 SUPPL. 1): S592-S593). 56th Annual Meeting of the American Society for Radiation Oncology, San Francisco, USA.	Conference proceeding
Lindner D.S. (2015). Managing breast and ovarian cancer risk: A novel approach to teaching residents comprehensive risk reduction and management strategies. Cancer Research **75**(9 SUPPL. 1). 37th Annual CTRC-AACR San Antonio Breast Cancer Symposium, San Antonio, USA.	Conference proceeding

Merriman J., et al. (2017) Breast cancer risk assessment and chemoprevention use among VA primary care. Journal of Clinical Oncology **35**(15 SUPPL. 1). 2017 Annual Meeting of the American Society of Clinical Oncology, USA.	Conference proceeding
Ozanne E., et al. (2011). Automated breast cancer risk assessment: Identifying high risk women in the primary care setting. Cancer Research **71**(24 SUPPL. 3). 34th Annual CTRC-AACR San Antonio Breast Cancer Symposium, San Antonio, USA.	Conference proceeding
Phillips K.-A., et al. (2013). Assessing breast cancer risk in primary care: What can we learn from cardiovascular disease?. Journal of Clinical Oncology **31**(15 SUPPL. 1). 2013 Annual Meeting of the American Society of Clinical Oncology, Chicago, USA.	Conference proceeding
Phillips K.-A., et al. (2018). Acceptability and usability of iPrevent, a web-based decision support tool for assessment and management of breast cancer risk. Cancer Research **78**(4 SUPPL. 1). San Antonio Breast Cancer Symposium, USA.	Conference proceeding
Schellenberg A., et al. (2018). Practitioner opinion on contralateral prophylactic mastectomy: How do we steer a patientdriven discussion?. Annals of Surgical Oncology **25**(2 SUPPL. 1): 94-95. 19th Annual Meeting of the American Society of Breast Surgeons, USA.	Conference proceeding
Smith S., et al. (2016). General practitioner attitudes towards prescribing tamoxifen for the primary prevention of breast cancer: Results of a vignette study. European Journal of Surgical Oncology **42**(11) (pp S233-S234). Joint BASO-ACS Annual Scientific Conference and NCRI Cancer Conference 2016, UK.	Conference proceeding
Stringhetta A. (2018). Recognizing histories: The knowledge of health professionals about hereditary cancer - a preliminary. International Journal of Gynecology and Obstetrics **143**(SUPPL. 3): 925-926. 22nd FIGO World	Conference proceeding

Congress of Gynecology and Obstetrics, Brazil.	
Vandezande L., et al. (2018). Genetic testing for breast cancer: Optimizing care for patients and their healthcare workers Development of a theoretical framework. European Journal of Cancer **92**(SUPPL. 3): S34. 11th European Breast Cancer Conference, Spain.	Conference proceeding
Wang H., et al. (2016). Breast cancer chemoprevention in primary care: Assessing readiness for change. Journal of Clinical Oncology **34**(SUPPL. 15). 2016 Annual Meeting of the American Society of Clinical Oncology, USA.	Conference proceeding
Zhang J.J., et al. (2018). Survey of physician decision-making and attitudes on preventive care priorities. Journal of General Internal Medicine **33**(2 SUPPL. 1): 351). 41st Annual Meeting of the Society of General Internal Medicine, USA.	Conference proceeding
Archer, S., et al. (2020). Evaluating clinician acceptability of the prototype CanRisk tool for predicting risk of breast and ovarian cancer: A multi-methods study. PLoS ONE, **15**(3): e0229999.	Ineligible exposure<br>*Clinicians' appraisals of a breast cancer risk assessment tool*
Battaglia, T. A., et al. (2006). Cancer prevention trials and primary care physicians: Factors associated with recommending trial enrollment. Cancer Detection and Prevention **30**(1): 34-37.	Ineligible exposure<br>*Assessed physician knowledge and attitudes regarding chemoprevention trials*
Brédart, A., et al. (2018). Use of the BOADICEA web application in clinical practice: appraisals by clinicians from various countries. Familial Cancer **17**(1): 31-41.	Ineligible exposure<br>*Clinicians' appraisals of a breast cancer risk assessment tool*
Bryan, T. J., et al. (2015). Impact of an educational intervention on provider knowledge, attitudes, and comfort level regarding counseling women ages 40–49 about breast cancer screening. Journal of Multidisciplinary Healthcare, **8**: 209-216.	Ineligible exposure<br>*Focuses on secondary prevention (breast screening)*

Colombet, I., et al. (2003). Combining risks estimations and clinical practice guidelines in a computer decision aid: a pilot study of the EsPeR system. Studies in Health Technology and Informatics **95**: 525-530.	Ineligible exposure<br>*Clinicians' appraisals of a multi-disease risk assessment tool*
Colombet, I., et al. (2003). A computer decision aid for medical prevention: a pilot qualitative study of the Personalized Estimate of Risks (EsPeR) system. BMC Medical Informatics and Decision Making **3**(1): 1-11.	Ineligible exposure<br>*Clinicians' appraisals of a multi-disease risk assessment tool*
de Bock, G. H., et al. (2001). How women with a family history of breast cancer and their general practitioners act on genetic advice in general practice: prospective longitudinal study. BMJ **322**(7277): 26-27.	Ineligible exposure<br>*Women's compliance with advice provided by their general practitioner and comparison of GP and clinical geneticists' advice*
De Bock, G. H., et al. (1999). GPs' management of women seeking help for familial breast cancer. Family Practice **16**(5): 463-467.	Ineligible exposure<br>*Management strategies for previous clinical cases*
Eden, K. B., et al. (2020). Use of an online breast cancer risk assessment and patient decision aid in primary care practices. Journal of Women's Health **29**(6): 763-769.	Ineligible exposure<br>*Clinicians' appraisals of a breast cancer risk assessment tool*
Emery, J., et al. (1999). Computer support for recording and interpreting family histories of breast and ovarian cancer in primary care (RAGs): qualitative evaluation with simulated patients. BMJ **319**(7201): 32-36.	Ineligible exposure<br>*Clinicians' appraisals of a breast cancer risk assessment tool*
Finkelstein, J., et al. (2017). Introducing a comprehensive informatics framework to promote breast Cancer risk assessment and chemoprevention in the primary care setting. AMIA Summits on Translational Science Proceedings: 58-67	Ineligible exposure<br>*Focuses on development of informatics framework to increase breast cancer risk assessment and chemoprevention*
Glasspool, D. W., et al. (2001). Risk assessment in genetics: A semi-quantitative approach. Studies in Health Technology and Informatics **84**(Pt 1): 459-463	Ineligible exposure<br>*Focuses on development of genetic risk decision support tool*

Hidalgo, K. D., et al. (2016). Health promoting practices and personal lifestyle behaviors of Brazilian health professionals. BMC Public Health **16**(1): 1114.	Ineligible exposure<br>*Health promotion practices for breast cancer patients*
Ka'ono'I, M. E., et al. (2004). Primary care physicians' knowledge, attitudes and practices related to cancer screening and cancer prevention clinical trials. Pacific Health Dialog **11**(2): 160-165.	Ineligible exposure<br>*Focuses on secondary prevention (breast cancer screening) and cancer prevention in general*
Lane, D. S., et al. (2001). An educational approach to improving physician breast cancer screening practices and counseling skills. Patient Education and Counseling **43**(3): 289-301.	Ineligible exposure<br>*Focuses on secondary prevention (breast screening)*
Lo, L. L., et al. (2018). The iPrevent online breast cancer risk assessment and risk management tool: usability and acceptability testing. JMIR Formative Research **2**(2): e24.	Ineligible exposure<br>*Clinicians' appraisals of a breast cancer risk assessment tool*
Nguyen, M. N., et al. (2009). Quebec breast cancer screening program: A study of the perceptions of physicians in Laval, Que. Canadian Family Physician **55**(6): 614-620.	Ineligible exposure<br>*Focuses on secondary prevention only (breast screening and clinical breast examination)*
Brownson, R. C., et al. (1993). Cancer control knowledge and priorities among primary care physicians. *Journal of Cancer Education* **8**(1): 35-41.	No breast cancer specific data<br>*Focuses on cancer risk and primary prevention whereby data specific to breast cancer cannot be extracted*
Carroll, J. C., et al. (2016). Primary care providers' experiences with and perceptions of personalized genomic medicine. Canadian Family Physician **62**(10): e626-e635.	No breast cancer specific data<br>*Focuses on experiences with, perceptions of, and desired role in personalized medicine, with a focus on cancer. Data specific to breast cancer cannot be extracted*
Flynn, B. S., et al. (2010). Primary care physicians' use of family history for cancer risk assessment. BMC Family Practice **11**(1): 45.	No breast cancer specific data<br>*Reports a confidence outcome but data specific to breast cancer cannot be extracted*

Kurashi, N. Y. (2007). Public health care physicians' knowledge, attitudes and management about breast cancer. Public Health Medicine **6**(2): 61-67.	No breast cancer specific data<br>*Knowledge, attitudes and practice outcomes but none about breast cancer risk assessment or primary prevention*
Mouchawar, J., et al. (2001). Colorado family physicians' knowledge of hereditary breast cancer and related practice. Journal of Cancer Education **16**(1): 33-37.	No breast cancer specific data<br>*Behaviour outcome is not breast cancer specific (do you collect family history information as part of your routine practice?)*
Tessaro, I. A., et al. (1996). Cancer prevention knowledge, attitudes, and clinical practice of nurse practitioners in local public health departments in North Carolina. Cancer Nursing **19**(4): 269-274.	No breast cancer specific data<br>*Attitudes & clinical practice outcomes but not specific to breast cancer*
Wilkes, M. S., Day, F. C., Fancher, T. L., McDermott, H., Lehman, E., Bell, R. A., & Green, M. J. (2017). Increasing confidence and changing behaviors in primary care providers engaged in genetic counselling. BMC Medical Education **17**: 163.	No breast cancer specific data<br>*Self-efficacy and attitudes outcomes but not specific to breast cancer*
Douma, K. F., et al. (2016). Non-genetic health professionals' attitude towards, knowledge of and skills in discussing and ordering genetic testing for hereditary cancer. Familial Cancer **15**(2): 341-350.	No breast cancer specific data<br>*Attitudes outcomes but not specific to breast cancer*
Teng, I., & Spigelman, A. (2014). Attitudes and knowledge of medical practitioners to hereditary cancer clinics and cancer genetic testing. Familial Cancer **13**(2): 311-324.	No breast cancer specific data<br>*Attitudes & clinical practice outcomes but not specific to breast cancer*
Ahmad, S., et al. (2011). Knowledge, attitude and practice for breast cancer risk factors and screening modalities in staff nurses of Ayub Teaching Hospital Abbottabad. Journal of Ayub Medical College Abbottabad **23**(3), 127-129	Ineligible population<br>*Author confirmed that participants did not provide primary care services in line with the World Health Organisation's definition*
Alkhasawneh, I. M. (2007). Knowledge and practice of breast cancer screening among Jordanian nurses. Oncology Nursing Forum **34**(6): 1211-1217.	Ineligible population<br>*Author confirmed that participants did not provide primary care services in line with the World Health Organisation's definition*

Javed, M., et al. (2013). Knowledge, attitude and preventive practices for breast cancer among health professionals at Shalamar medical and Dental College/Hospital Lahore. Pakistan Journal of Medical & Health Sciences **7**(56): 582-587.	Ineligible population<br>*Participants worked at a tertiary care private teaching hospital*
Karayurt, Ö., et al. (2010). Evaluation of the breast cancer train the trainer program for nurses in Turkey. Journal of Cancer Education **25**(3): 324-328.	Ineligible population<br>*Author confirmed that participants did not provide primary care services in line with the World Health Organisation's definition*
Panic, N., et al. (2014). Survey on knowledge, attitudes, and training needs of Italian residents on genetic tests for hereditary breast and colorectal cancer. BioMed Research International **2014**.	Ineligible population<br>*Author confirmed that participants did not provide primary care services in line with the World Health Organisation's definition*
Prolla, C. M. D., et al. (2015). Knowledge about breast cancer and hereditary breast cancer among nurses in a public hospital. Revista Latino-Americana de Enfermagem **23**(1): 90-97.	Ineligible population<br>*Author confirmed that participants did not provide primary care services in line with the World Health Organisation's definition*
Welkenhuysen, M., & Evers-Kiebooms, G. (2003). Predictive genetic testing for breast cancer and Huntington's disease: the opinions of midwives and nurses in Flanders. Public Health Genomics **6**(2): 104-113.	Ineligible population<br>*Author confirmed that participants did not provide primary care services in line with the World Health Organisation's definition*
Collins et al. (2014). Assessing and managing breast cancer risk: Clinicians' current practice and future needs. The Breast **23**(5):644-650	Qualitative methodology<br>*For reasons of space, the review was limited to quantitative findings only*
Donnelly et al. (2020). Implementing risk stratification into the national breast cancer screening programme: perspectives of NHS personnel responsible on feasibility and impact. Unpublished	Qualitative methodology<br>*For reasons of space, the review was limited to quantitative findings only*
Phillips, K.-A. et al. (2016). Transitioning to routine breast cancer risk assessment and management in primary care: what can we learn from cardiovascular disease? Australian Journal of Primary Health **22**(3): 255-261.	Qualitative methodology<br>*For reasons of space, the review was limited to quantitative findings only*

Smith et al. (2016). Clinician-Reported Barriers to Implementing Breast Cancer Chemoprevention in the UK: A Qualitative Investigation. Public Health Genomics, **19**(4): 239-249.	Qualitative methodology<br>*For reasons of space, the review was limited to quantitative findings only*
Brown, J., et al. (2019). Physician knowledge, attitudes, and practices regarding breast density. Journal of Women's Health **28**(9): 1193-1199.	Data pertinent to PCPs could not be extracted<br>*Author contacted to clarify sample size of PCPs who confirmed that PCPs & geriatricians were grouped for analysis therefore it is not possible to extract the findings relevant to the PCPs*
Entrekin, N. M., & McMillan, S. C. (1993). Nurses' knowledge, beliefs, and practices related to cancer prevention and detection. Cancer Nursing **16**(6): 431-439.	Data pertinent to PCPs could not be extracted<br>*Staff nurses recruited from multiple settings so findings relevant to those working in primary care cannot be extracted*
Gabram, S. G., et al. (2009). Assessing breast cancer risk and providing treatment recommendations: immediate impact of an educational session. The Breast Journal **15**(s1): S39-S45.	Data pertinent to PCPs could not be extracted<br>*PCPs and surgeons recruited; it is not possible to extract the findings relevant to the PCPs*
Igid, H., et al. (2019). Breast Cancer Risk Assessment and Evaluation of Risk-Based Screening Practices by Primary Care Providers: A Single Institution Experience. Clinics in Oncology **4**: 1625.	No data from perspective of PCPs<br>*Retrospective chart review*
Owens, W. L., et al. (2011). Implementation in a large health system of a program to identify women at high risk for breast cancer. Journal of Oncology Practice, **7**(2): 85-88.	No data from perspective of PCPs<br>*Implementation only*
Ozanne, E. M., et al. (2009). Identification and management of women at high risk for hereditary breast/ovarian cancer syndrome. The Breast Journal **15**(2): 155-162.	No data from perspective of PCPs<br>*Implementation only*
Baker, S. K. (2016). Rural Arizona nurse practitioners' knowledge of hereditary breast and ovarian cancer risk assessment (Publication No.	Insufficient data reported

10245174) [Doctoral dissertation, The University of Arizona]. ProQuest Dissertations Publishing.	*Eligible data but not sufficiently reported to include in review. No correspondence email address as it is a doctoral dissertation. Search performed on Scopus but no email address identified for the author*
Yadav, S., et al. (2019). Utilization of a breast cancer risk assessment tool by internal medicine residents in a primary care clinic: impact of an educational program. BMC Cancer **19**: 228.	Insufficient data reported<br>*Eligible data but not sufficiently reported to include in review. Author contacted three times for additional data but no response received*
Hunter, A., et al. (1998). Physician knowledge and attitudes towards molecular genetic (DNA) testing of their patients. Clinical Genetics **53**(6): 447-455.	Insufficient data reported<br>*Eligible data but not sufficiently reported to include in review. Corresponding author unable to provide data so forwarded query to co-author but no response was received*
Harris, H., et al. (2011). Familial breast cancer: is it time to move from a reactive to a proactive role?. Familial Cancer **10**: 501-503.	Insufficient data reported<br>*Eligible data but not sufficiently reported to include in review.*<br>*Author contacted and relevant data sent. Unable to stratify responses by country so data provided did not resolve percentage error in paper*
Yaren, A., et al. (2008). Awareness of breast and cervical cancer risk factors and screening behaviours among nurses in rural region of Turkey. European Journal of Cancer Care **17**(3): 278-284.	Unclear population<br>*Author contacted three times for clarity on population but no response received*
Korde, L. A., & Gadalla, S. M. (2009). Cancer risk assessment for the primary care physician. Primary Care: Clinics in Office Practice **36**(3): 471-488.	Overview article<br>*Not primary research*
